# Modular microfluidics enables kinetic insight from time-resolved cryo-EM

**DOI:** 10.1038/s41467-020-17230-4

**Published:** 2020-07-10

**Authors:** Märt-Erik Mäeots, Byungjin Lee, Andrea Nans, Seung-Geun Jeong, Mohammad M. N. Esfahani, Shan Ding, Daniel J. Smith, Chang-Soo Lee, Sung Sik Lee, Matthias Peter, Radoslav I. Enchev

**Affiliations:** 1 Institute of Biochemistry, Department of Biology, ETH Zurich, Otto-Stern-Weg 3, 8093 Zurich, Switzerland; 2 The Visual Biochemistry Laboratory, The Francis Crick Institute, 1 Midland Road, NW1 1AT London, UK; 3 Department of Chemical Engineering and Applied Chemistry, Chungnam National University, Yuseong-Gu, Daejeon, 305-764 Republic of Korea; 4 Structural Biology Scientific Technology Platform, The Francis Crick Institute, 1 Midland Road, NW1 1AT London, UK; 5 Scientific Center for Optical and Electron Microscopy, ETH Zurich, Otto-Stern-Weg 3, 8093 Zurich, Switzerland

**Keywords:** Structural biology, Biophysics, Electron microscopy, Cryoelectron microscopy, Engineering

## Abstract

Mechanistic understanding of biochemical reactions requires structural and kinetic characterization of the underlying chemical processes. However, no single experimental technique can provide this information in a broadly applicable manner and thus structural studies of static macromolecules are often complemented by biophysical analysis. Moreover, the common strategy of utilizing mutants or crosslinking probes to stabilize intermediates is prone to trapping off-pathway artefacts and precludes determining the order of molecular events. Here we report a time-resolved sample preparation method for cryo-electron microscopy (trEM) using a modular microfluidic device, featuring a 3D-mixing unit and variable delay lines that enables automated, fast, and blot-free sample vitrification. This approach not only preserves high-resolution structural detail but also substantially improves sample integrity and protein distribution across the vitreous ice. We validate the method by visualising reaction intermediates of early RecA filament growth across three orders of magnitude on sub-second timescales. The trEM method reported here is versatile, reproducible, and readily adaptable to a broad spectrum of fundamental questions in biology.

## Abstract (web summary)

There is a growing interest in performing time-resolved cryo-EM studies. Here, the authors present a time-resolved sample preparation method for cryo-EM called trEM, which uses a microfluidic device to initiate the biochemical reaction by rapid mixing of the components and then spraying the sample onto a cryo-EM grid to snap-freeze it in a blot-free, automated manner within several milliseconds.

## Introduction

The structures of biological macromolecules largely determine biochemical activity, mechanism, and specificity through highly dynamic conformational changes. This remodelling is coupled to functions such as enzyme catalysis, allostery, and interactions with other macromolecules, typically occurring on microsecond to millisecond timescales^1,2^. Such structural rearrangements proceed through energetically accessible transition-states^3^ without destabilizing the overall fold necessary for function^4^. Early studies demonstrated that proteins exist in a variety of conformational sub-states that correspond to local energy minima^5^. However, it is experimentally challenging to isolate these states for structural studies and relate them to functionally relevant intermediates.

Cryo-electron microscopy (cryo-EM) and single-particle analysis are widely used to determine structures of biological macromolecules^6^. One distinct advantage of the method is that the samples are prepared in solution under nearly native conditions, allowing the imaging of numerous instances of individual specimens and application of statistical methods to disentangle compositional and conformational variability. Indeed, recent studies have shown that many separate states can be identified and solved to high resolution within a cryo-EM sample^4,7^. However, such workflows do not directly identify functionally relevant states, nor do they elucidate the sequence in time of structural transitions underpinning a reaction pathway. Moreover, short-lived active intermediates, which are rare under equilibrium conditions, remain undetected. Therefore, there has been a long-standing interest in developing time-resolved cryo-EM sample preparation and analysis to visualise such states. First successes came from cryo-electron crystallography^8–12^ by diffusing small interactants into crystals, broadly analogous to time-resolved X-ray crystallography techniques^13^ and limited to crystalline samples and small diffusible ligands. More recent attempts in the framework of cryo-EM and single-particle analysis have traced structural changes over time in processes that proceed over several seconds and even minutes. This was only possible because the studied processes were slower than the time required to prepare a cryo-EM sample grid by the standard method of manual application, followed by automated blotting and plunge-freezing^14–17^. Dynamic structural states could also be resolved by freeze-trapping samples, pre-incubated at different temperatures^18^. However, both of these techniques are applicable to a small subset of biochemical reactions that have slow kinetics or show substantial temperature sensitivity, and do not enrich short-lived intermediates.

To overcome these limitations and develop a general time-resolved sample preparation method for cryo-EM (trEM) requires building a miniaturized mixer and bioreactor able to rapidly initiate and synchronize biochemical reactions, followed by spreading the incubated sample onto a cryo-EM grid without the need for manual operation or blotting, collectively faster than the lifetime of the structures of interest^19^. Previous work has shown that combining reactants in microfluidic devices followed by rapid application of sample by gas-assisted spraying is in principle possible^20–25^, and has yielded fascinating new insights into biology^26^. However, substantial technical challenges remain unaddressed. For instance, in order to widen the applicability of the method, developments are needed to reduce the complexity of microfluidics design and manufacturing without diminishing the reliability and reproducibility of results. Additionally, gas-assisted aerosol generation for blot-free sample application through a microfluidic device has not been studied systematically. Lastly, the ability to achieve reliable mixing on millisecond timescales inside microfluidic devices has not been experimentally assessed in the context of trEM, nor have the errors on nominal in-chip incubation times been measured. Collectively these pose major impediments to conducting well-controlled time-resolved structural studies by cryo-EM, as have been reported by time-resolved X-ray crystallography and free electron laser experiments^27^.

Here we address most of the above by developing a trEM workflow that incorporates a modular microfluidic platform with an in situ 3D mixer and tunable incubation time from ten to thousands of milliseconds, capable of enriching intermediate states of biochemical reactions on cryo-EM grids. The system is largely automated and simple to manufacture without access to specialised facilities. Comprehensive assessment of the obtained cryo-EM sample quality demonstrated the unique advantages of rapid blot-free sample vitrification for sample integrity and uniform distribution across the grid. Moreover, we established a biochemical model system, derived from the bacterial homologous recombination pathway, which allowed us to characterise the obtainable time-resolution in quantifiable terms. We demonstrate the ability to extract kinetic information from structural changes on timescales spanning three orders of magnitude pertinent to macromolecular function. Combining all the above developments we present a robust methodology for the study of fragile and dynamic structures by trEM.

## Results

### An integrated system for time-resolved preparation of cryo-EM samples

**Fig. 1:**
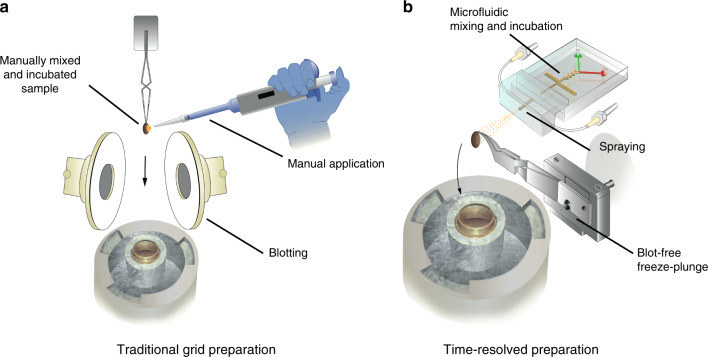
Sample preparation methods for cryo-EM. **a** Drawing of the standard method, which entails manually pipetting several microliters of sample onto a support grid, removing the excess liquid by blotting (touching with filter paper) and vitrification by plunge-freezing into liquid ethane. The most popular commercial system for this preparation is marketed as Vitrobot, and this name is used interchangeably with standard cryo-EM sample preparation throughout the text. **b** Drawing of time-resolved cryo-EM sample preparation. A microfluidic device mixes and incubates a biochemical reaction, then sprays the sample onto a plunging grid such that blotting is not required, and finally the sample grid is plunged into liquid ethane to achive vitrification.

In standard cryo-EM sample preparation, several microliters of sample are pipetted onto a support grid, followed by removing most of the sample liquid with blotting paper (Fig. 1a). This leaves a very thin layer of sample that can be vitrified under ambient pressure and imaged with high contrast and minimal secondary scattering^28^. However, blotting requires at least a few seconds and is thus incompatible with time-resolved studies of most biochemical processes. The blotting process and its duration also likely cause denaturation of some fragile samples^29,30^. To prepare a cryo-EM sample on millisecond timescales, we developed an integrated sample preparation method with substantial automation (Fig. 1b). This system (Supplementary Fig. 1a) utilises microfluidic devices that rapidly and reliably mix and then incubate reactants on timescales relevant to the studied biochemical process. A gas-assisted nozzle generates a sample aerosol, which is applied, blot-free, onto a standard cryo-EM support grid and rapidly plunged into liquid ethane by a servo motor. The assembly is modular so that different microfluidic devices can be operated by the same set-up, ensuring high reproducibility. In addition, the system can be placed into an environmental chamber with humidity and temperature control. An integrated electronic board and software were developed to coordinate the function of all components (Supplementary Fig. 1b, c). This control system is instrumental to reduce sample volume, as it ensures constant flow for a minimal time before spraying the sample onto the grid. The design principles and optimised working specifications of each major component are described in detail below.

### Rapid microfluidic mixing to initiate biochemical reactions

**Fig. 2:**
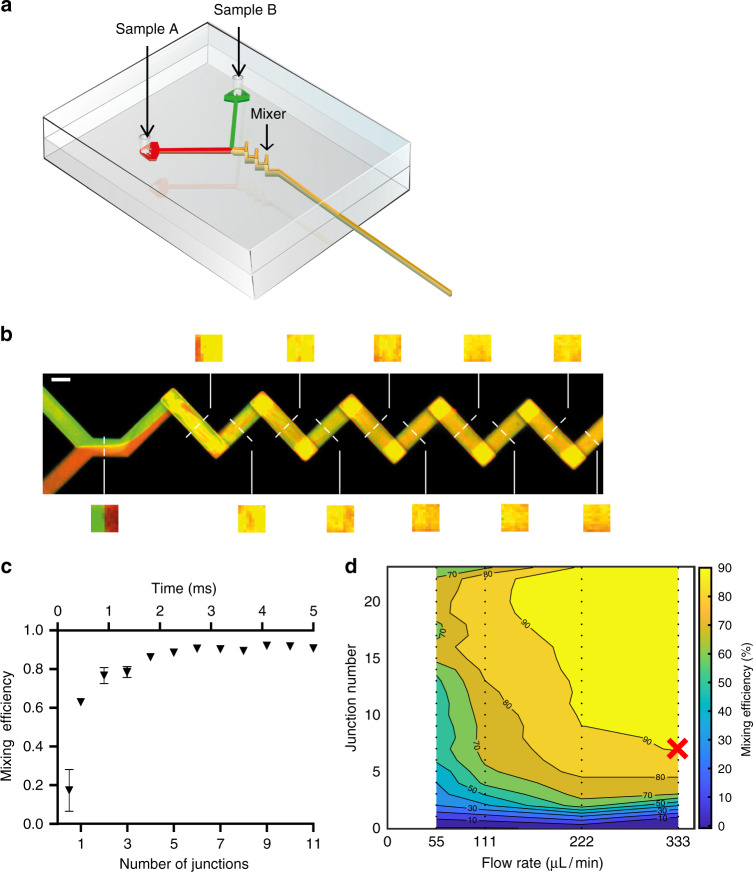
Rapid and reliable sample mixing in a microfluidic device. **a** Schematic representation of a microfluidic device with the mixer geometry shown in yellow. **b** Confocal micrograph representing an optical slice through the centre of a mixing microfluidic geometry at steady state flow, 333 μl/min per channel, of two fluorescent dyes. Transversal slice reconstructions through the channel used for quantification of the mixing efficiency are shown. Scale bar is 100 μm. **c** Quantification of mixing efficiency data shown in (**b**) as a function of 3D mixing elements (junctions) and time. Error bars represent standard deviation from three separate slices through the channel. Source data are provided as a Source Data file. **d** Phase diagram representation of mixing efficiency as a function of liquid flow rate (per channel) and number of 3D mixing elements. The condition minimising both flow rate and mixing channel length used in the remainder of this work is indicated (red cross).

The first critical element is the initiation of the biochemical reaction inside the microfluidic chip by mixing. Kinetic rates that determine the formation and lifetimes of reaction intermediates are dependent on the local concentration of the components^31,32^. Thus, to reliably enrich for a specific state at the endpoint of freezing, the starting point must be precisely defined by optimizing the mixing of the individual reactants. We adopted an in situ three-dimensional (3D) mixer, previously reported to significantly enhance mixing efficiency^33^ (Fig. 2a, Supplementary Fig. 2a). In the mixing region, the bulk fluid flow is along the axis of the channel but fluid around a channel bend has three-dimensional flow combined with secondary flows, generated in the channel cross-section. These secondary flows integrated with the axial flow distort and stretch interfaces and can produce chaotic advection. Thus, the interfacial area across which diffusion occurs is greatly increased, and enables near-perfect mixing in millisecond time and nanolitre volumes.

To validate the above theoretical consideration and to quantify the mixing efficiency and time, we developed a dedicated experimental workflow. We produced a microfluidic device composed of a large number of sequential 3D-mixing elements (junctions), pumped two distinguishable fluorophore solutions through the two channels and mounted it on a confocal microscope to image the mixing along the channel as a function of 3D junctions and flow rates (Fig. 2b, Supplementary Fig. 2c). The mixing efficiency and time were then determined by image analysis of cross-sections along the channel (Fig. 2c, Supplementary Fig. 2d). We observed a secondary flow-induced thinning striation across the channel with increasing number of junction and flow rates, resulting from a vigorous mixing of two discrete flows **(**Fig. 2b, Supplementary Fig. 2c). The sample flow rate and size of the mixing region were chosen to minimize the mixing time and required flow rate (Fig. 2d, Supplementary Fig. 2d).

To further validate this experimental set up, we performed an independent experiment whereby we pre-mixed the two fluorophores on a shaker for an extended period of time before injecting through the chip. The result was quantified as the other experiments and is plotted alongside them in Supplementary Fig. 2d. As expected, the value remains largely unchanged throughout the measurement, in contrast to the asymptotic behaviour observed for the mixing experiments at high flow rates. The data from the pre-mixed sample were more noisy, presumably due to some precipitation of the fluorophores, preventing us from using that dataset for normalization purposes.

Based on this analysis, we chose a flow rate of 333 μl/min per channel (total of 666 μl/min after merging the two channels) and seven mixing junctions for all experiments reported below, as it reached maximum mixing efficiency observed in the shortest amount of junctions. This also determined a total mixing time of 3.1 ms, as calculated from the fluid average velocity and physical length of the mixing junctions. To accurately calculate fluid velocity both the dimensions of the microfluidic channel (Supplementary Fig. 2b) and the performance of the fluid pump (“Methods”) were experimentally verified, finding only a few percent error from expected values.

### Rapid, blot-free cryo-EM sample preparation

**Fig. 3:**
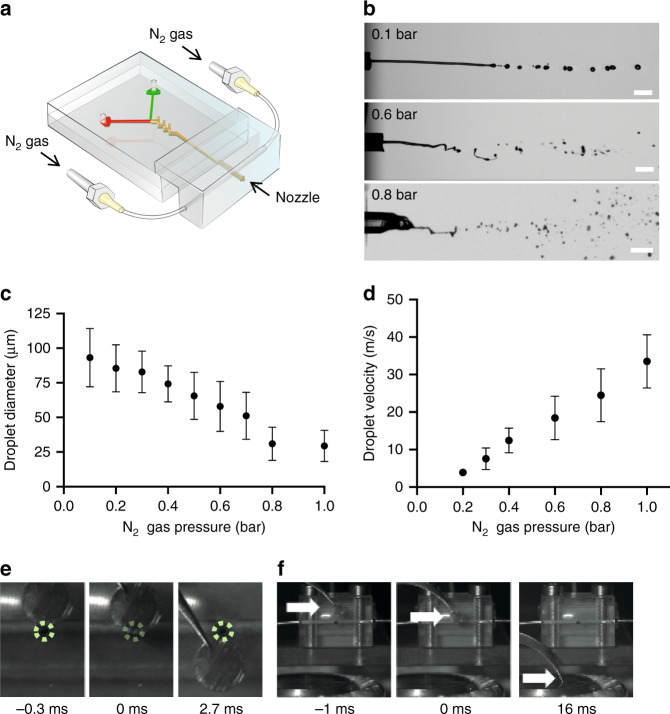
Sample spray and blot-free delivery onto a TEM support grid. **a** Schematic representation of a microfluidic device with a gas-assisted spraying nozzle. **b** Snapshots from high-speed camera datasets of sample sprayed at indicated nominal gas pressure. Scale bar is 363 μm. **c**, **d** Quantification of droplet diameters (**c**) and speed (**d**) as a function of increased gas pressure. Error bars represent standard deviation across the total population of droplets quantified. Source data are provided as a Source Data file. **e**, **f** Snapshots from high-speed camera imaging of a cryo-EM sample grid plunging (**e**) past the nozzle (nozzle position indicated in dotted green) and (**f**) from the nozzle into the liquid ethane container (positions indicated by white arrows), with time stamps in milliseconds.

After initiating the reaction inside the microfluidic device, the sample needs to be rapidly delivered to a cryo-EM support grid without compromising reaction synchronisation. To achieve this, we developed a gas-assisted nozzle (Supplementary Fig. 3a) that produces a fine sample aerosol applicable without the need for time-consuming blotting. The nozzle was tailored to the microfluidic geometry and liquid flow rate determined above, with sample flowing at 666 μl/min out of a 100-μm circular orifice. A concentric gas stream breaks the sample jet into small droplets and accelerates them towards the grid, which allows them to spread on impact into a thin enough film for cryo-EM imaging. The nozzle assembles modularly with the microfluidic chips (Fig. 3a), which allows integrating devices with distinct properties. Increasing the gas pressure produces smaller, albeit more irregular droplets (Fig. 3b, c) but also increased droplet velocity (Fig. 3d, Supplementary Movie 1, 2). Nitrogen gas pressure of 0.8 bar was found to be optimal to generate small enough droplets to spread into thin layers on the grid without significant damage to the support grid.

The next critical step was the collection of sample aerosol onto a grid and its subsequent vitrification. This was done by attaching a pair of tweezers to a servo motor and radially plunging the grid into a liquid ethane container at a tuneable speed (Fig. 1b, Supplementary Fig. 1a). Servo motors are affordable and easily customizable, for example by combining with gears to increase or decrease the plunging speed **(**Supplementary Data 10, 11). We found that plunging at 100 rpm (~1.1 m/s radial speed) at a distance of 8 mm from the nozzle consistently produced grids with a sufficient number of vitrified holes suitable for cryo-EM imaging and high resolution single-particle analysis. This results in an integration time of sample on the grid of 2.7 ms (Fig. 3e), and 16 ms between mixing of the sample to vitrification (Fig. 3f). Faster plunging was not found to be beneficial as it reduced the amount of sample collected on the grid, while as expected, slower plunging fails to vitrify the sample. These parameters define the preparation time to around 20 ms, along with an integration time of ~3 ms. The time of flight of the sample spray and subsequent vitrification was <2 ms^34^. Together with the 3 ms required for sample mixing (see above), the resulting dead time is around 30 ms, which is fast enough to approach many hitherto unstudied biological reaction timescales. When all tubing and microfluidic channels were filled with buffer prior to use, a sample volume of 20–30 μl per grid was necessary, since at least 2 s were found to be required for the flow to equilibriate and produce predictable spraying patterns.

### Assessment of cryo-EM sample prepared by blot-free spray-plunging

**Fig. 4:**
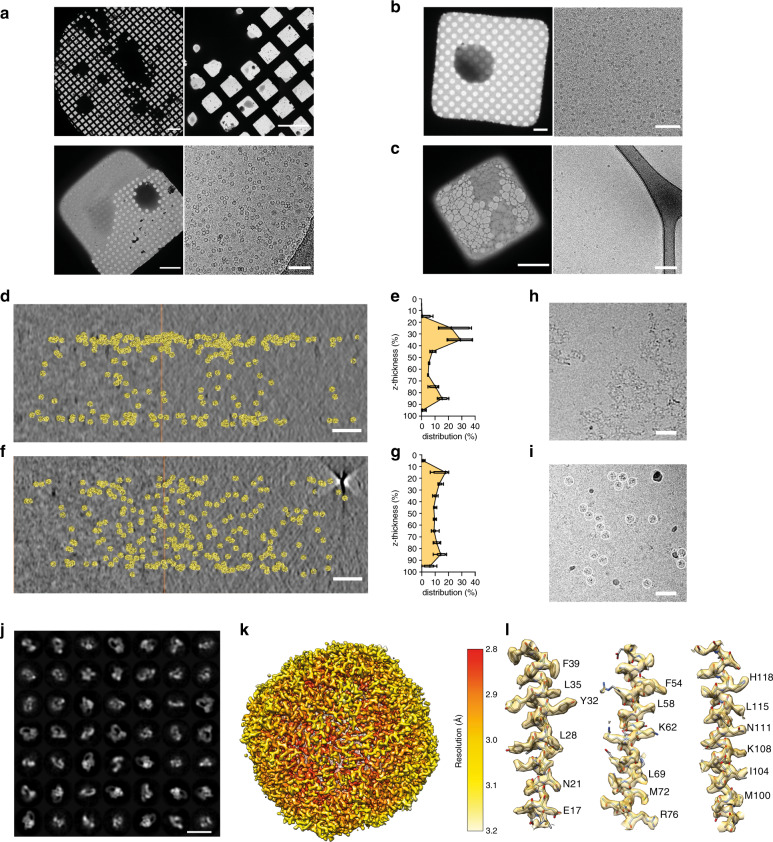
Assessment of cryo-EM samples prepared by blot-free spray-plunging. **a** Apoferritin sprayed on holey Quantifoil grid. Scale bars represent 200 μm in upper left panel, 100 μm in upper right panel, 10 μm in lower left panel, 50 nm in lower right panel. **b** CSN^5H138A^-SCF-N8^Skp2/Cks1^ complex^39^ on a Quantifoil grid covered with thin carbon. Scale bars are 10 μm in the left panel, and 100 nm in the right panel. **c** 20S proteasome^89^ on a lacey grid covered with thin carbon. Scale bars are 5 μm in left panel, and 10 nm in the right panel. **d**–**g** Tomographic reconstructions and segmentation of apoferritin distribution across the depth of a vitrous sample spanning a hole of a Quantifoil grid produced by the standard method (**d**) and blot-free spray-plunging (**f**). Scale bars are 50 nm. Quantification of the distribution of sample as a function of depth (in 10% steps) produced by conventional (**e**) and blot-free spray-plunging (**g**) from 15 independent measurements for each sample. Error bars represent standard deviation. Source data are provided as a Source Data file. **h**–**i** CSN^5H138A^-SCF-N8^Skp2/Cks1^ distribution on a holey Quantifoil grid using the standard method (**h**) and blot-free spray-plunging (**i**). Single particles in (**i**) are indicated with circles. In **h**, the protein is only in an aggregated state. Scale bars 50 nm. **j** Selected 2D classes of CSN^5H138A^-SCF-N8^Skp2/Cks1^ from (**i**). Scale bar 20 nm. **k** Single-particle reconstruction of apoferritin from a sample prepared by blot-free spray-plunging at a global resolution of 2.77 Å, local resolution indicated by the given colour code. **l** Representative fit of atomic model and density of indicated regions.

We applied trEM sample preparation to a series of different commercially available sample support grids and biological samples to assess the overall quality of ice and amount of microscopically collectable areas (Fig. 4a–c). At the optimised plunging speed and distance from the nozzle to grid described above, we routinely observed sufficient numbers of grid squares containing sample droplets with thin ice. We detected the most thinly spread droplets over holey Quantifoil grids, however Quantifoil and lacey grids purchased with an ultrathin continuous layer of carbon also contained droplets that yielded micrographs with high-contrast vitrified single particles. (Fig. 4b, c). To ascertain the reproducibility of the above results, we sampled over 20 individual grids for each type. In all cases we observed many grid squares with thin ice, typically averaging between 30 and 75 grids squares with ~600–800 collectable holes. Since we preferentially used holes with a 2-μm diameter, these could afford up to four micrographs per hole on a Titan KRIOS with an electron beam condensed to 1 μm under data collection conditions, usually enough for collecting a large enough dataset from a single grid. The particles were observed to be unperturbed, albeit with a similar but slightly lower concentration of protein compared to the standard procedure, as also noted in other rapid sample application methods^35^. For the remainder of this work, we focus on the most widely used grid type for cryo-EM, holey Quantifoil.

To assess the sample quality obtained by this method we first generated tomographic reconstructions to determine the protein distribution relative to the air-water interfaces^29,30,36^. We used apoferritin as a model protein and compared biochemically identical samples prepared by trEM to the standard technique (Fig. 4d–g, Supplementary Fig. 4a). The image contrast is comparable to the standard technique, although the average ice thickness was somewhat greater than 100 nm. To facilitate direct comparison, we collected tomograms from regions of matching ice thickness on grids prepared using a Vitrobot. Consistent with previous reports^30,37,38^ we observed a pronounced bi-phasic distribution of sample due to clustering at the air-water interfaces when prepared on a Vitrobot (Fig. 4d) among multiple areas of independently produced grids (Fig. 4e). Importantly, following the same analysis and quantifications of sample grids prepared by trEM we detected a significantly more uniform distribution of protein along the z-direction (Fig. 4f, g), indicating an important improvement to sample quality.

To further characterise the quality of the sample, we collected apoferritin datasets from both preparation methods for high-resolution structure determination by single-particle analysis. Datasets of similar size were collected and subjected to an identical single-particle analysis workflow (Supplementary Fig. 4B, c, Table 1). We were able to confirm that high-resolution structure of apoferritin could be reconstructed from data obtained by trEM grid preparation and that all expected near-atomic resolution details were retrieved (Fig. 4k, l). Importantly, the comparative analysis showed no significant difference between the datasets, with similar numbers of particles picked, similar proportions retained at each round of processing, and similar final resolution achieved (Supplementary Fig. 4B, c). We thus conclude that the established trEM workflow reliably produces high-quality cryo-EM samples on sub-second timescales.

**Table 1:** Cryo-EM data collection and refinement statistics.

	Vitrobot apoferritin (EMDB-10714)	Sprayed apoferritin (EMDB-10712)
*Data collection and processing*		
Magnification	96,000	96,000
Voltage (kV)	300	300
Electron exposure (e–/Å^2^)	33.6	33.6
Defocus range (μm)	−0.5 to −2.5	−0.5 to −3.5
Pixel size (Å)	0.845	0.845
Symmetry imposed	o	o
Initial particle images (no.)	146,581	133,833
Final particle images (no.)	19,485	9804
Map resolution (Å)	2.89	2.77
FSC threshold	0.143	0.143
Map resolution range (Å)	2.84–3.15	2.82–3.19
2D classification	99,271 particles after two rounds of 2D classification	65,202 particles after three rounds of 2D classification
3D classification	19,485 particles after one round of 3D classification	9804 particles after one round of 3D classification

Since apoferritin is not known to be prone to denaturation or aggregation during cryo-EM sample preparation, we performed further trEM experiments with purified CSN^5H138A^-SCF-N8^Skp2/Cks1^ complex^39^. We previously studied its structure using holey Quantifoil grids overlaid with a thin carbon support^39^, as it aggregates over open holes when prepared on a vitrobot (Fig. 4h). Interestingly, using rapid blot-free grid preparation, we observed monodisperse complexes over the holes of Quantifoil grids without a thin carbon film (Fig. 4i). 2D classification of the particles confirmed that the sample is structurally intact (Fig. 4j)^39^, suggesting that the rapid trEM preparation method improves the molecular integrity of sensitive samples.

### Trapping pre-steady state kinetic intermediates by cryo-EM

**Fig. 5:**
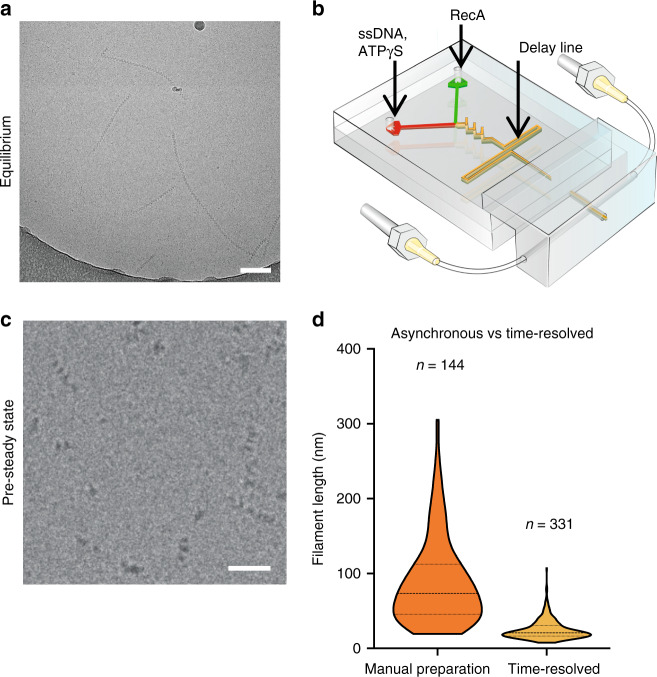
Observation of RecA-ssDNA filament growth by cryo-EM. **a** RecA filaments on a grid prepared using the standard method. Scale bar is 100 nm. **b** Diagram of a mixing chip with a delay line. **c** RecA filaments observed on a grid prepared by blot-free spray-plunging in a chip with a 20-ms delay line. Scale bar is 50 nm. **d** Violin plot of quantified filament lengths from (**a**) and (**c**). Dashed line indicates the median filament length, and the shape of the violin curve indicates proportion of filaments at different lengths. Source data are provided as a Source Data file.

To assess the fidelity of synchronization, and the ability to visualise pre-steady state reaction intermediates by trEM, we sought a biochemical test sample with broad significance to biology, and well-understood kinetics and steady-state structure, which would be readily correlatable in cryo-EM micrographs without recourse to novel image processing or quantification algorithms. We chose to study the prototypical bacterial recombinase RecA, which mediates homologous recombination pathways^40^ and is thus a critical regulator of DNA damage repair and antibiotic resistance arising from horizontal gene transfer. In particular, we probed the known kinetic behaviour of ATPγS-dependent growth of E. coli RecA protein on single-stranded DNA. RecA-ssDNA helical filaments are simple to observe in raw electron microscopy micrographs^41^ (Fig. 5a) and the length of these filaments is directly related to the reaction incubation time. Their growth kinetics have been studied in detail on both short and long time scales^42–44^. Following a rate-limiting nucleation step by a cluster of 2–6 RecA monomers, filaments are rapidly extended by first-order reaction kinetics by sequential monomer addition^44^. In the presence of ATPγS, saturation of the oligonucleotides with RecA results in juxta-position of neighbouring filaments, resulting in concatenation^45–47^ and the appearance of longer filaments than would be expected from the contour length of a single oligonucleotide fully decorated with RecA. Moreover, the length of filaments on cryo-electron micrographs is easily quantifiable with basic image analysis tools.

To evaluate the ability to kinetically trap and image biochemical pre-steady state intermediates, as opposed to steady-state equilibrium structures, we performed parallel experiments. To this end, RecA was mixed with single-stranded DNA (ssDNA) and ATPγS, either manually or inside of a microfluidic mixing chip (Fig. 5b), followed by cryo-EM grid preparation. For the manual mixing and grid preparation, the incubation was several minutes and resulted in an equilibrium of different filament lengths (Fig. 5a, d). For the reaction incubation inside a chip we adapted the microfluidic concept that path length inside the device determines the incubation time, provided that velocity of the fluid is tightly controlled^48^. Thus, we introduced a delay line downstream of the mixing region, resulting in a nominal in-chip incubation time of around 20 ms (Fig. 5c, d, Supplementary Fig. 5A, b). We observed nucleated RecA filaments at the earliest time points of filament formation. As expected, observed RecA filament lengths were much shorter (Fig. 5c), and the length distribution was markedly reduced compared to the manual preparations (Fig. 5d). The asynchrony in lengths following manual preparation is easily understandable due to the fact that observed filaments have reached the equilibrium of provided reactants and are no longer undergoing first-order filament extension.

The deviation from the mean in the microfluidic experiments, albeit much smaller, merits further attention. One source of experimental inaccuracy in microfluidics may arise from the interaction of the reactants with the surfaces of the chip. To measure this effect, we quantified the adsorption of protein, DNA, and ATP following passage through PDMS microfluidic channels^49^ (Supplementary Fig. 5c). We observed differential interactions of the reactants, where 19% of protein, 29% of DNA, and 42% of ATP were adsorbed by PDMS during a one-second incubation. Variance in adsorption is likely due to different diffusivity and electrostatic properties, which are expected to vary slightly depending on the reactants used^50^. Nevertheless, these values would not result in rate-limiting depletion of the reactants. The slow nucleation of RecA filaments generates some overall error in reaction initiation as a proportion of filaments will only start growing after they have travelled an uncertain length along the delay line. Thus many filaments of shorter length than the average likely originated from such late nucleation events.

In contrast, the outliers towards larger than average length filaments likely arise due to the fluid dynamics of the system. The Reynolds numbers inside the used microfluidic chips under steady-state flow conditions are in the range of 100, resulting in laminar flow throughout the microfluidic channels, with the exception of the mixing region. This results in slower flow rates closer to the edges of the channel. Thus, the in-chip residence time, equivalent to incubation in a biochemical reaction, is characterised by a non-Gaussian distribution instead of a sharp peak which is, on average, equal to the nominal time but tends to be significantly longer for a subpopulation of reactions. Indeed, computational fluid dynamics simulations (Supplementary Fig. 5d) and particle tracing predicted a characteristic residence time distribution as expected by the Taylor dispersion in laminar flow^51,52^ (Supplementary Fig. 5e). These results are in good overall agreement with the observed filament length distribution (Fig. 5d) and suggest that errors due to residence time scattering in the microfluidic chip are likely to be the dominant source of inaccuracies in time-resolution experiments conducted with this or similar systems.

### Pre-steady state RecA-ssDNA filament kinetics determined by trEM

**Fig. 6:**
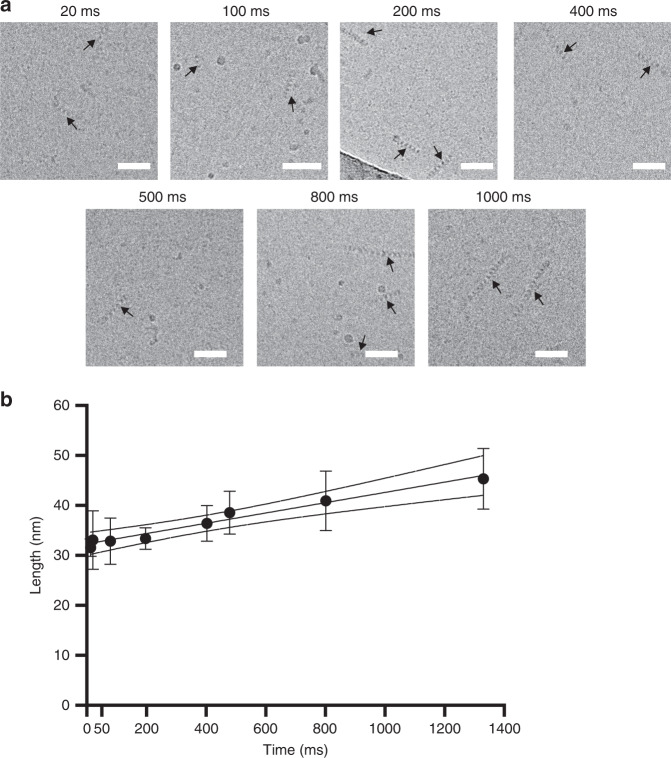
Kinetic analysis of RecA-ssDNA filament growth by time-resolved cryo-EM. **a** Example micrograph of each studied time point indicated in milliseconds. Scale bars are 50 nm. **b** Growth curve quantifying the averages of the median lengths of at least three replicates per time point, shown are standard deviations (error bars) and the 95% confidence interval of the linear regression fit. R-squared of the fit through the means of medians is 0.973. Source data are provided as a Source Data file.

Finally, having thoroughly characterised the scope of trEM operational parameters and their limitations, we designed and performed a proof-of-concept experiment aimed at obtaining kinetic information solely through image analysis of pre-steady state structural changes visualised by trEM. We performed a time-course of RecA filament growth reactions on sub-second timescales spanning three orders of magnitude to determine the fidelity of final time-resolution. This was done by constructing a series of microfluidic devices with varying lengths of the incubation channel that defines specific reaction times (Supplementary Fig. 6a). In each chip, purified RecA was mixed with ssDNA and ATPyS, and the reactions sprayed onto EM grids. Each timepoint of RecA filament growth was captured in at least three independent experiments, on separately prepared reaction mixtures on different days to test for the reproducibility of the method. The length of RecA filaments was then measured at each time point by cryo-EM analysis with, on average, several hundreds of individual filaments per replicate (Fig. 6a, b). By determining median filament length per replicate, we obtained linear growth rates for RecA-ssDNA filaments of roughly ~20 nt/s, which closely matches previously reported growth rates observed over orders of magnitude longer time scales^42^. This was done by assuming that each RecA monomer holds three DNA bases and extends the filament by 1.53 nm^42^. This showed that we could robustly resolve pre-steady state growth of RecA filaments at the earliest timepoints using only structural information as a readout (Fig. 6a, Supplementary Fig. 6b). Moreover, there was little experimental variation for each time point (Supplementary Fig. 6b). These results quantify the fidelity of time-resolution achievable by trEM, and demonstrate that the developed time-resolved cryo-EM method is robust and broadly applicable.

## Discussion

Structural biology provides highly detailed 3D models of biological macromolecular assemblies. However, the dynamic conformational changes required to perform specific functions often remain unobserved, emphasising the need to develop methods that can time-resolve biochemical reactions as they occur, without sacrificing molecular detail. Exploring such structural transitions, especially the intermediates that enable function, will provide a new window into understanding the very basics of biochemistry. In this study, we present a major step in the development of time-resolved structural studies, by developing a reliable and robust methodology for the initiation, synchronisation, and freeze-trapping of biochemical reactions onto cryo-EM sample support grids for subsequent analysis.

There have been other developments of time-resolved cryo-EM methods^22,23,26,53–55^, including a very recently reported adaptation of existing technology, Spotiton, for time-resolved studies^56^. Some devices already enabled outstanding contributions to resolving unknown biochemistry^26,54,55^, however important steps remain to be improved for the general usability of trEM. We designed an integrated system with a focus on versatility, alongside the charactierisation of all components and ascertaining reproducibility for broadly adopting this method. We utilised widely available materials and processes to produce modular microfluidic devices, enabling mixing and incubation of biological macromolecules, and interfacing with a gas-assisted spraying nozzle for rapid blot-free cryo-EM sample preparation. We quantified both the mixing and the droplet sprays across a wide range of operating parameters and performed detailed assessement of the potential losses of sample which can occur inside the microfluidic devices. Alongside careful delienation of the dead time and quantification of the fluid dynamics effects, collectively offer a complete description of the achievable time resolution. In contrast we show that humidified chambers, intricate gas-mixing systems, and voltage-assisted spraying are not necessary to obtain high quality micrographs using spray-based sample delivery. Importantly, we characterised the compatibility of this set up with a range of the most popular commercially available cryo-EM support grids (Fig. 4a–c). Lastly, since many biochemical events often take place across time scales spanning several orders of magnitude from single-digit milliseconds to several seconds, the presented methodology that enables capture of all these timepoints. Indeed, our device provides multi-order time resolution from 10–1000 ms, and in principle longer. We made a series of modular microfluidic devices with different residence times of 10, 20, 80, 200, 400, 480, 800, 1330 ms and attachable slip-on gas nozzles for the ease of utilization. To demonstrate the multi-order time-resolution, we quantified progression of a biochemical reaction in RecA growth on ssDNA and obtained reliable biochemical growth rates for RecA-ssDNA filaments, on a par with biophysical growth rates determined over longer timescales^42,44,45^. Moreover, we used trEM to examine apoferritin, 20 S proteasomes and CSN^5H138A^-SCF-N8^Skp2/Cks1^ complexes. Importantly, we found that the short grid residence time before vitrification improves structural integrity while the ice thickness was not the limiting factor for final resolution, as has been the case with other methods. Using trEM sufficient micrographs for high-resolution reconstructions can be collected from a single grid, produced with approximately 30 μl of sample, providing good throughput of data. Taken together, the described trEM method provides a robust and integrated system that successfully combines biochemical and structural studies.

Kinetic data obtained by trEM sample preparation and image analysis has the key advantage of not relying on fluorescent or radioactive labels. All reactants used in the microfluidic experiments were free in solution with no outside intervention or specific release mechanisms. This demonstrates that de novo biochemical reaction rates can be determined using fixation by vitrification of specific timepoints along the reaction path. A further major advantage of this technology is the ability to holistically study all structural transitions in the same experiment, rather than monitoring areas close to the chosen label sites, as done in routinely established biophysical methods. Future experiments will be designed to exploit these new opportunities, for example by performing time-resolved studies to elucidate the catalytic mechanism of CSN-mediated CRL deneddylation^39^ and the specificity determinants of homologous recombination^57^, and will undoubtedly greatly broaden the applications and biological insights obtainable by trEM.

The automated system for time-resolved cryo-EM sample preparation described here (Fig. 1b, Supplementary Fig. 1a) is versatile, simple to build and modify, and readily adaptable to different experimental needs. We have given considerable attention to practical details, and all technical drawings and code to build the device are made available. Notably, the replica-molding of PDMS, in comparison to previously reported etched silicon devices, ensures reproducibility in microfabrication and minimises cross-contamination. The entire design and production process relies solely on affordable and readily available components and techniques, without the need to access clean room facilities, making it possible to implement this time-resolved system in most biological research institutions and thus widen its applications. Its two major features are a rapid blot-free sample vitrification method using a gas-assisted nozzle to generate a sample spray, and a tunable microfluidic mixing and incubation delay line, which can be readily customised to cover the vast majority of timescales relevant to biochemical processes.

The standard and most widely used method of cryo-EM sample preparation relies on manual sample application with a pipette, followed by blotting of excess liquid and vitrification of the resultant thin film (Fig. 1a)^28^. Although transformative for structural biology, this method also has limitations. For instance, the longer the sample spends in a thin film prior to vitrification, the more likely it is to contact the air-water interfaces potentially leading to denaturation^29^ or to adopt a preferred orientation, resulting in loss of structural information due to lack of viewing angles^58^. Recent improvements to the support grids and to how the sample is applied have begun to address these issues^29,35,59,60^. Here we describe a different approach, using standard grids rapidly plunging through a sprayed, atomised sample (Fig. 1b, Supp Movie 1), faster than 30 ms. We determined a near-atomic resolution structure by single-particle analysis of an apoferritin sample prepared in this way (Fig. 4k, l and Supplementary Fig. 4b), which was practically indistinguishable from one obtained by standard preparation (Supplementary Fig. 4c). However, the apoferritin particle distribution inside of the thin vitreous layers was markedly different between the two methods. Consistent with previous reports of rapid sample application onto self-wicking grids^36^, we demonstrated a close to completely uniform distribution for the rapidly prepared sample (Fig. 4f, g), as opposed to more than half the particles being stuck to the interfaces following standard preparation (Fig. 4d, e). Importantly, the sprayed sample spent less than 3 ms on the carbon grid before vitrification, compared to time spent using conventional methods. Future work will further clarify the relative contributions of blotting and preparation times. While apoferritin does not suffer from denaturation at the surface of the ice or from preferred orientations, the CSN^5H138A^-SCF-N8^Skp2/Cks1^ complex^39^ is strongly denatured at the air-water interface (Fig. 4h) and no cryo-EM structures of this or related complexes have been reported using holey grids. However, using trEM sample preparation, we were able to obtain sample distribution on the grid that was monodisperse (Fig. 4i) and with its native conformation preserved, as indicated by 2D class averages (Fig. 4j). Importantly, we expect that this method will facilitate structural studies of other samples that are adversely affected by the air–water interfaces.

A second major limitation of the standard cryo-EM sample preparation method is that the process is too slow to capture intermediate states with lifetimes of milliseconds without the aid of mutagenesis, chemical inhibitors or crosslinking agents. To circumvent this limitation, we approached two further challenges. The first was rapid and complete mixing to initiate a biochemical reaction. Although diffusion equilibrates the concentrations of sample components over time, on average proteins diffuse <100 μm/s, which prevents the synchronisation of biochemical reactants on sub-second time scales^61^. In the field of microfluidics, this problem has been successfully solved by the use of active or passive mixers which ensure complete mixing in milliseconds^62–64^. In this work, we incorporated a passive mixing geometry (Fig. 2a) and experimentally determined the optimal combination of 3D mixing elements and flow rate to achieve near-complete mixing in about 3 ms (Fig. 2c). Together with the blot-free sample application by spraying onto an accelerated plunging grid (Fig. 3b, f), this defines the total dead time of our method to be 30 ms, which ensures its applicability to studying many biochemical processes (Supplementary Fig. 3b).

Having effectively established rapid and reliable mixing to define the starting point of a reaction, the second challenge was assessing the synchronisation over the course of the experiment inside the microfluidic delay line (Fig. 5b). Importantly, time-resolved growth of RecA filaments experimentally demonstrated significant enrichment of a characteristic length distribution at all studied time points (Fig. 6, Supplementary Fig. 6b), implying that the current setup allows a label- and fixation-free method to study transient intermediates. Optimal synchronization is governed by the residence time distribution (RTD) of the microfluidic geometry at the specific flow condition. We assessed the RTD under our experimental conditions by computational fluid dynamics (CFD) simulation analysis (Supplementary Fig. 5C, d) and found that our simulations reliably predict experimental outcomes (Fig. 5c, d). RTD arises physically from the laminar flow in microfluidic channels at steady state, where due to dead zones in the geometry the reactants can incubate for an indefinite time before exiting^65^. This can generate overlap in populations, potentially complicating subsequent analysis. Since the RTD comprises the biggest limitation to achievable time resolutions, it is worthwhile considering stop-flow systems and geometries that could reduce its effects. An alternative approach could be to accurately determine the extent of RTD through CFD simulations and experimental model systems and treat it as a linear response function of the system, attempting correction by deconvolution.

The major advances reported here as well as by others^21,26^ prompt further meaningful improvements to trEM. Currently, the amount of sample consumed per experiment is large in comparison to other sample preparation approaches^35,66,67^. Due to the use of a microfluidic mixing and incubating device, continuous flow is required, which in turn necessitates several seconds of flow before reaching a steady operating condition when the flow is generated by a syringe pump. Using injection valves to maintain steady flow without unnecessarily spraying sample could in principle reduce sample consumption to sub-microliter volumes. The analysis of the droplet sizes and speeds generated by our nozzle (Fig. 3c, d) suggest a promising additional improvement—the use of gas-dynamic virtual nozzles^68–70^. These nozzles are capable of producing droplet jets with similar parameters at much lower fluid flow rates, further reducing sample consumption.

Apart from the above engineering challenges of improving time-resolved sample preparation for cryo-EM, single-particle analysis of pre-steady state datasets harbours particular difficulties. Multiple structurally similar states may co-exist and it will be critical to detect them reliably in order to quantify their relative populations at any studied time point, thus establishing the order of molecular events. Unbiased algorithmic mining of conformational heterogeneity by emerging techniques will be of paramount importance^7,71–74^.

The experimental approach to preparing time-resolved samples for cryo-EM and single-particle analysis enabled us to reliably and reproducibly obtain near-atomic spatial and two-digit millisecond temporal resolution. Furthermore, we demonstrate the ability of trEM to closely follow biochemical reactions by using the model system of RecA filament growth, obtaining kinetic rates from raw micrographs that are in agreement with existing biophysical data. Thus, since most major difficulties in adapting cryo-EM to time-resolved studies have been circumvented and/or characterised, we are confident that time-resolved cryo-EM will offer unprecedented insights into biochemical reactions, including the sequence of reaction intermediates and pathway stochasticity.

## Methods

### Manufacturing the cryo-EM grid preparation setup

The frame of the setup was manufactured from stainless steel in a standard mechanical workshop. A transparent plastic box encasing the entire set up was also custom made and connected to a humidifier (UHW 60065, Medisana). Modified N7 tweezers (Dumont) were attached through a custom-made mount to a servo motor (HT750, Highest Korea). The fluid pump was a syringe pump (PHD Ultra, Harvard Apparatus). Two hundred and fifty microlitres of gas-tight glass syringes (1700 PTFE Luer Lock, Hamilton) were used with the pump. Arduino mega 2560 (Arduino) controls the position of the tweezer in the rotation arm, on/off of the spraying gas valve, and the humidifier. Positioning between spraying nozzle and tweezers was done with XYZ stage (BO9-47, Suruga Seiki). The grid was plunge-frozen in a standard vitrobot ethane/nitrogen container (ThermoFischer – part no. FEI0815S).

### Electrical board and software integration/control

The electronics board (Supplementary Fig. 1B, c) was manufactured to apply correct voltage for both power and control to each component, as well as containing an option for a second servo, with voltage control between 7.5 and 5 V. Software control of the system was achieved by communication between the open-source electronic prototyping platform (Arduino) and a PC. The Arduino was running the Firmata library, allowing other PC software to relay commands to the Arduino. The actual control software was a simple user-friendly GUI written in Processing (Supplementary Data 1), using the controlP5 library for visual control elements. The pump was controlled directly from Processing using a serial control library.

### High-speed camera imaging and data analysis

High-speed camera imaging was done with Fastcam Mini AX200 (Fig. 3e, f) or Nova S12 type 1000 K (Fig. 3b, Supplementary Movie 1, 2) (Photron) and a LED light source (F5100, Photonic) and quantified using VisiSize (Oxford Lasers). Lenses used were a 12× lens with fixed focus, 2× adapter, 0.5× and 2× attachment lenses (Navitar). Camera control was achieved by the manufacturer’s software (PFV4, Photron). LED light was directly behind the imaged area for optimal illumination, when needed. Standard imaging settings for sprays were 100,000 frames per second, 0.2 μs shutter speed, 1024 × 128 pixel resolution (Fig. 3b). For droplet landing imaging 80,000 frames per second, 0.2 μs shutter speed, 384 × 304 resolution (Supplementary Movie 1, 2). For imaging tweezers plunging, we used 10,000 frames per second, 33.3 μs shutter (due to inability to position light directly behind), 1024 × 1024 resolution (Fig. 3e, f). Movies were processed by applying software normalization and pixel gain in PFV software prior to imaging. Postprocessing with High-Dynamic Range (HDR) was used for grid landing movies to compensate for the obstructed illumination caused by the grid passing between the camera and the illumination source.

### PDMS chip design and manufacturing

The nominal fluid velocity and therefore channel geometry per timepoint was calculated using the Poiseuille formula for frictional pressure drop in laminar flow, adapted for rectangular channels. For this purpose, a publicly available web-based microfluidic calculator (Dolomite Microfluidics) was used. Times were rounded to the nearest 10 ms in Fig. 6b, Supplementary Fig. 6b.

Wafers for all types (Supplementary Data 2–9) of microfluidic devices were manufactured using photolithography with UV exposure on negative photoresist via UV aligner (MA8, Suss MicroTec) or a direct photolithography machine (MicroWriter ML3, Durham Magneto Optics) on 3” silicon wafers (PI-KEM - WAFER-SILI-0004W25). SU-8 2035 photoresist (Microchem) was spin-coated onto the wafer at 1055 rpm for 30 s to reach a desired thickness of 100 μm (±3 μm). All wafers were quality controlled by optical depth profiling using the optical profiler using interferometry with a 10× objective (S neox, Sensofar Metrology). Chips were designed such that 3D mixing features were formed by alignment of the top and bottom layers of the PDMS channel. For replica molding, the conventional soft lithography technique with polydimethylsiloxane (PDMS, Sylgard 184, Dow Corning) mixture was applied. The PDMS was mixed 10:1 base to curing agent ratio, mixed for 10 min, degassed under vacuum, and poured onto the wafer. After hardening by baking at 110 degrees, the chips were cut out and bonded after plasma treatment for 30 s. After plasma activation both sides of the chip were aligned and pressed together under a microscope by hand. After baking the bonded chips overnight at 80 degrees, fused silica tubing (TSP 100375, BGB) was inserted and glued with transparent silicone glue (Elastosil E43, Wacker) at the outlet to form the exit tubing, which inserts into the nozzle. We verified the output flow rate from the pump and the microfluidic chip by collecting the output flow for 1 min. Independently produced PDMS chips were measured 10 times by weighting the collected outflow and comparing to the expected output volume.

### RecA filament growth reactions

RecA filament growth buffer contained 25 mM Tris-HCl (pH 7.5), 10 mM magnesium-acetate, 100 mM sodium-acetate, 1 mM DTT. Final reaction mixture also contained 1 mM ATP-γ-S, 10.57 μM RecA (NEB - M0249L) (0.4 mg/ml), 31.71 μM ssDNA (3-fold of RecA). The ssDNA used was 144-mer (TTTTTTCTCACACTCATTTTTTTTCTCACACTCATTTTTTTTCTCACACTCATTTTTTTTCTCACACTCATTTTTCCTATATTTATTCCTTTTCCTATATTTATTCCTTTTCCTATATTTATTCCTTTTCCTATATTTATTCCT). Reactions were performed at room temperature. For filament growth experiments, two different solutions were prepared. One contained buffer, ATP-γ-S and ssDNA the other contained buffer and RecA. These were loaded into glass syringes and then inserted in the microfluidic chip through silica tubing (TSP 100375, BGB) to initiate the reaction. All concentrations stated are final concentrations after mixing inside the device. Quantifications of filament lengths were done in FIJI software^75^, by manually tracing filament lengths.

### Mixing quantification by fluorescence microscopy

Tile scans of the entire mixing channel were acquired using a Leica SP8 confocal microscope (pin hole at 1AU) over 300 μm height (61 z-slices, every 5 μm), using a 10×/0.4 objective (Leica HC PL APO CS2) with final pixel size being 1.0111 μm. Fluorophores used were FITC and Rhodamine 6G, excited with 488 nm and 561 nm laser lines respectively in sequential mode to minimize the cross-talk. Before imaging, the fluorophores flowed in the channel for 30 s to reach a steady-state flow. After every junction, the z-stack was resliced along the z-axis to obtain transversal cross sections images (re-slice made with FIJI software^75^) (Fig. 2b). We analysed a region of 10 × 10 pixels at the centre of the channel to avoid artefacts from the channel walls. Quantification of mixing efficiency was done by comparing the cross section at the start of the channel to the ones after each junction, measuring the mixing index in terms of standard deviation of pixel values, using equations previously described^75^.

### Cryo-EM grid preparation

Quantifoil R2/2, holey, 300 mesh grids were glow discharged for 40 s at 40 mA with a K100X glow discharger (EMS). For freezing a Vitrobot Mark IV (FEI ThermoFisher) was used at room temperature and 100% humidity. Four microlitres of of apoferritin (diluted to 2 mg/ml in PBSA, pH 7.4) (Sigma - A3641) was pipetted onto the grid, blotted for 3 s, and plunge-frozen.

### Time-resolved cryo-EM grid preparation

Grids were glow discharged for 40 s at 40 mA using a K100X glow discharge device (EMS). Grids were held at a distance of 8 mm from the nozzle. Sample was sprayed at 666 μl/min of sample flow and 0.8 bar nitrogen gas. Plunging speed was roughly 1.1 m/s. Grids used in experiments: Quantifoil R2/2, 2 nm Carbon, 400 mesh (Fig. 4b). Quantifoil R2/2, holey, 300 mesh (Fig. 4a, d, f, h, i and high-resolution datasets). Lacey carbon film with ultrathin carbon 400 mesh (Agar Scientific) was used in Fig. 4c.

### Single-particle analysis

High-resolution cryo-EM data were acquired on Titan Krios operated at 300 keV with a FalconIII detector in counting mode with 20 frames per movie and nominal magnification of 96000× (0.845 Å pixel size). The electron dose was 33.6 e/Å^2^. For the sprayed apoferritin dataset the defocus range was −0.5 to −3.5 μm, while the vitrobot apoferritin dataset had a range of −0.5 to −2.5 μm. The larger defocus was due to the slightly thicker ice in sprayed samples. Movies were motion-corrected by using MotionCor2 with 5 by 5 patches^76^ and CTF estimation was done by CTFfind4 on dose-weighted micrographs^77^. All particle picking was done by crYOLO using a model trained in-house^78^. Subsequent image processing was performed in Scipion and RELION-3, using PDB 1AEW^79^, filtered to 40 Å as a reference^80,81^. CSN^5H138A^-SCF-N8^Skp2/Cks1^ complexes were purified as described^39,82^, and data for 2D classification collected on a Talos Arctica (Thermo-Fisher Scientific, Waltham, MA) operating at 200 keV and with a Falcon III direct electron detector in linear mode with 10 frames per movie, 6.5 e/Å^2^, 1.61 Å pixel size. Movies were aligned using MotionCor2 and CTF estimation was done by gCTF^83^. Particle picking was done by crYOLO using an in-house trained model^78^ followed by 2D classification in cryoSPARC 2.14.2^84^.

### Tomography of particle distributions

Tilt series were collected in Tomography 4.0 on a Talos Arctica (Thermo-Fisher Scientific, Waltham, MA) operating at 200 keV and with a Falcon III direct electron detector. The tilt series were collected in linear mode with a range of ±54 degrees and 3 degree increment at a pixel size of 5.18 Å/px. Each exposure received a dose of 3 e/Å^2^ for a total dose of 111 e/Å^2^. The defocus used was −8 μm. Tomograms were aligned in IMOD^85^ using 10 nm gold fiducials and reconstructed with weighted back-projection followed by median-filtering and down-sampling by a factor of four.

Template-matching of tomograms was performed with MolMatch software^86^. A low-pass filtered apoferritin map at 20 Å resolution was used as a template that was systematically translated across the tomogram before calculating the normalized cross-correlation with the tomographic volume. This procedure yielded a composite map containing the maximum cross-correlation coefficients (CCC) for each voxel in the tomogram. The top ~2000 CCCs and their 3D coordinates were extracted from the map using the AV3 toolbox in Matlab (av3_createmotl)^87^. After plotting the CCCs, extremely high (false positives) or low (false negatives) values were excluded with an empirically determined cut-off threshold. Any remaining false positives and negatives were manually pruned using the EM Package for Amira 5.3^88^ (Thermo-Fisher Scientific, Waltham, MA). The remaining ferritin matches were displayed in Amira 5.3 and their corresponding Z-coordinates were used for quantification. To facilitate comparison, we binned the z-height of each tomogram into 10-percentile bins and counted the total number of apoferritin particles located per bin across all tomograms to produce the plots and quantifications in Fig. 4e, g.

### Simulations of residence time distribution

Simulations were performed using COMSOL Multiphysics 5.4. Laminar flow simulations were performed with 3D models of the microfluidic device’s geometry. These were generated in Solidworks Professional Research 2019 (Solid Solutions) and simulation meshes were subsequently generated by using COMSOL built-in mesh generation tools using the fluid-dynamics preset. The stationary solution of the flow profile was then used as input, described by the Navier-Stokes equation, into a time-dependent study of particle tracking in the geometry. Proteins were approximated by 10 nm solid spheres with the molecular weight of 10 RecA monomers (378 kDa). Both drag forces from the flowing liquid and Brownian motion of particles were modelled as part of RTD analysis. Quantification was done by storing the total residence time of each particle inside the geometry from the inlet, measured at the outlet. Graphs were generated in Prism 8 using the “high” level of violin plot smoothing.

### Reporting summary

Further information on experimental design is available in the Nature Research Reporting Summary linked to this paper.

## Supplementary information

Supplementary Information

Description of Additional Supplementary Files

Supplementary Data 1

Supplementary Data 2

Supplementary Data 3

Supplementary Data 4

Supplementary Data 5

Supplementary Data 6

Supplementary Data 7

Supplementary Data 8

Supplementary Data 9

Supplementary Data 10

Supplementary Data 11

Supplementary Movie 1

Supplementary Movie 2

Reporting Summary

## Data Availability

EM maps are deposited in the EM Data Bank under EMD-10712 (trEM) and EMD-10714 (Vitrobot). Technical drawings and code are uploaded as Supplementary Data files. Source data are provided with this paper. Other data are available from the corresponding authors upon reasonable request.

## Source data

Source Data
