# Supplementary material for: Modular microfluidics enables kinetic insight from time-resolved cryo-EM
Source: Nat Commun. 2020 Jul 10;11:3465. doi: 10.1038/s41467-020-17230-4 (PMC7351747; doi:10.1038/s41467-020-17230-4)
Supplement: Supplementary file 1 — Supplementary Information [file 41467_2020_17230_MOESM1_ESM.pdf]

Modular microfluidics enables kinetic insight from time-resolved cryo-EM

Märt-Erik Mäeots, Byungjin Lee et al. 2020

Supplementary information

Supplementary Figures

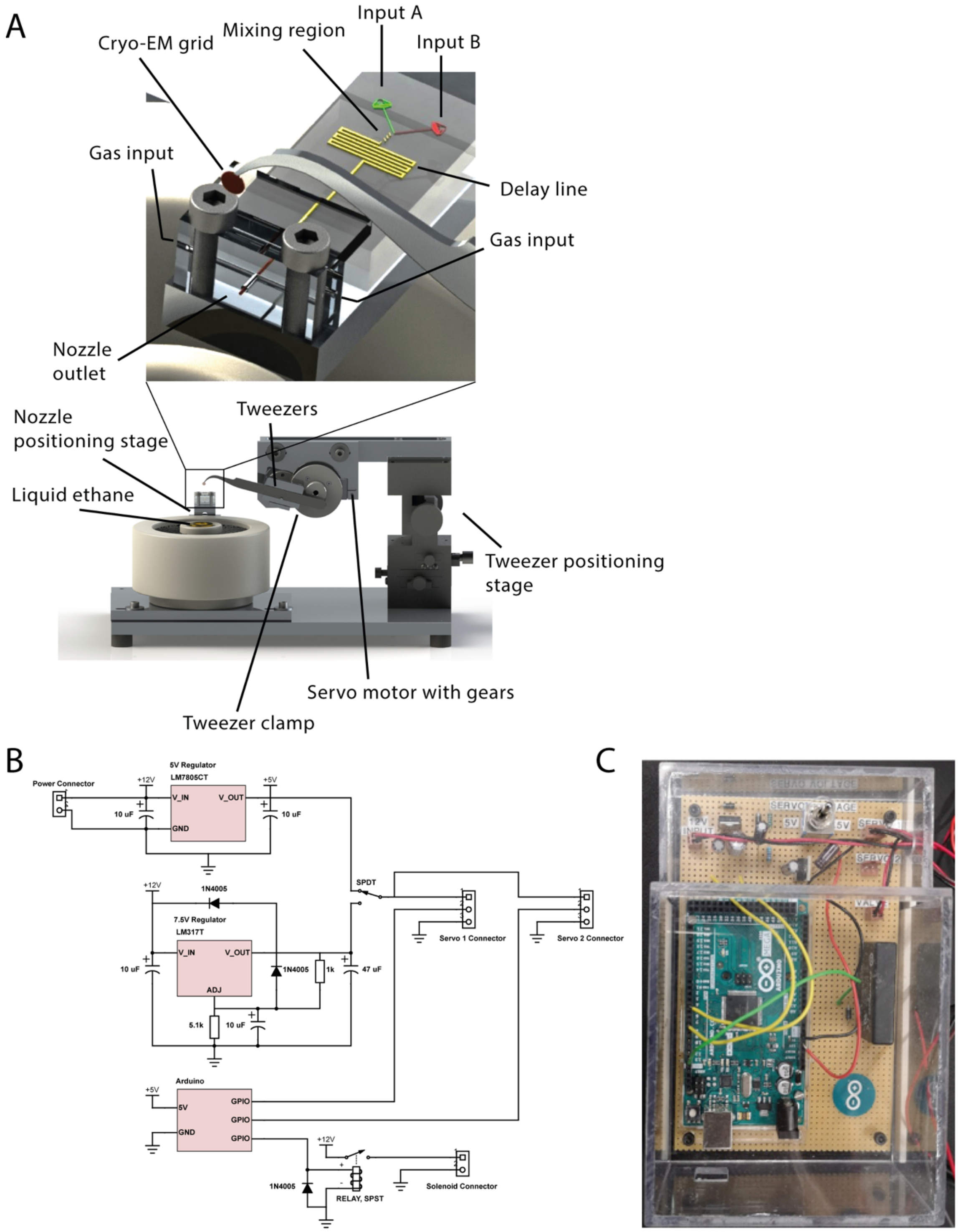

Supplementary Figure 1.

A Three-dimensional technical rendering of the set up for time-resolved cryo-EM sample preparation, indicating key elements. **B** Diagram of the electrical control board. It contains a voltage regulating circuit to manage power to different components as well as input/output from the Arduino. Not shown are the connections of the pump and camera, which connected directly via USB or ethernet to the Computer. **C** Image of the electronics board in a protective plastic case.

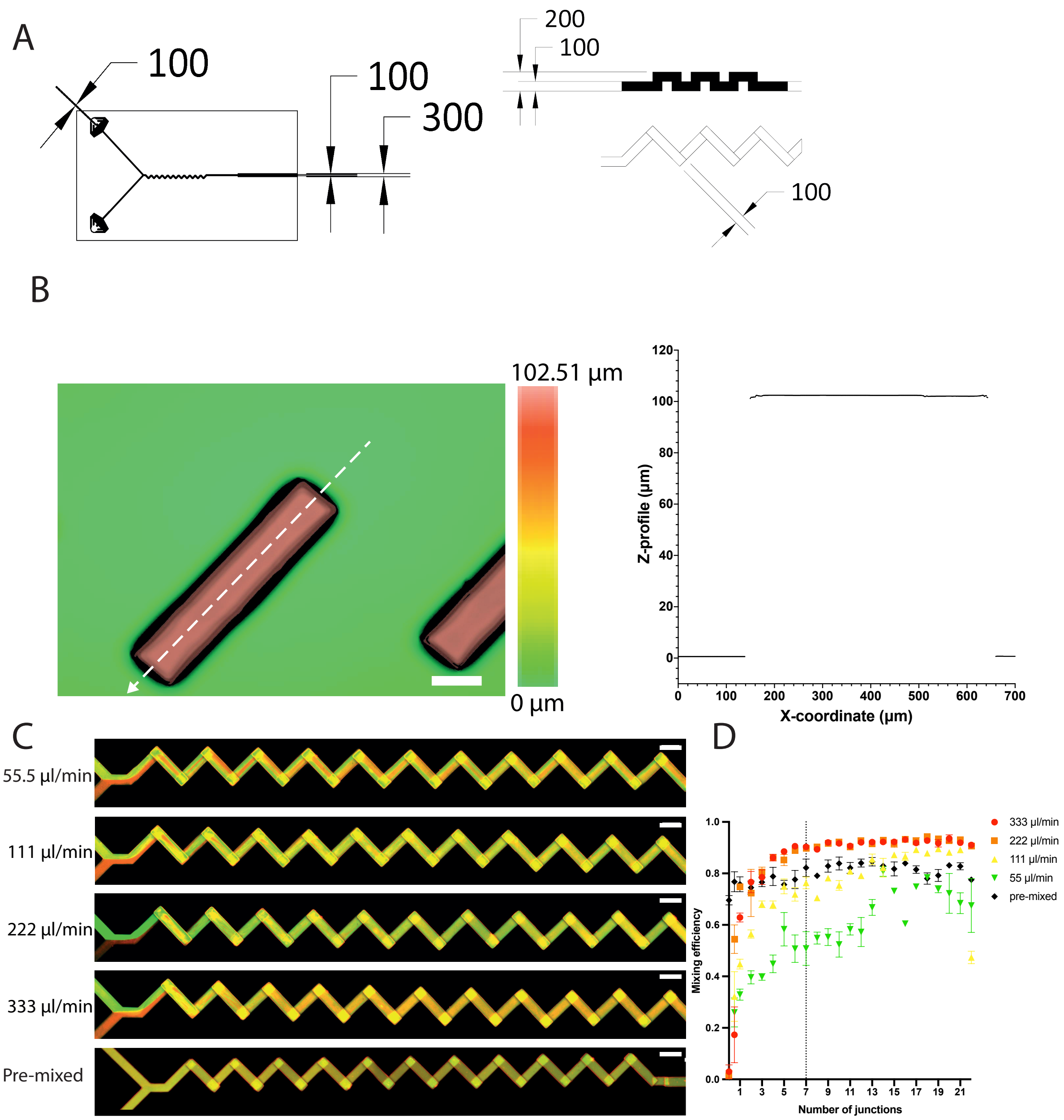

Supplementary Figure 2.

A Technical drawing of the overall microfluidic geometry and 3D passive mixing elements (left). Top view of overall microfluidic geometry, (right top) side view of 3D passive mixing element, and (right bottom) top view of the 3D passive mixing element. Dimensions in µm. **B** Depth profile of the wafer used to manufacture the PDMS devices. Left panel depicts region of measurement, white scalebar 100 µm in XY, color coding for Z-scale as shown. Right panel shows Z-profile along the the white dotted line, direction indicated by arrowhead. Mean height of the channel 101.67±0.18 µm. Black coloured areas could not be measured due to the sharp edges. Source data are provided as a Source Data file. **C** Confocal micrographs of mixing two fluorescent dyes at the indicated steady state flow rates. Scale bars are 200 µm. **D** Quantification of mixing efficiency data show in **C** as a function of 3D mixing junctions. Time to reach the 7th junction is 3.18 ms for 333ul/min, 4.73 ms for 222ul/min, 9.46 ms for 111 ul/min, and 18.92 ms for 55.5 ul/min. Error bars represent standard deviation from 3 separate slices through the channel. Source data are provided as a Source Data File.

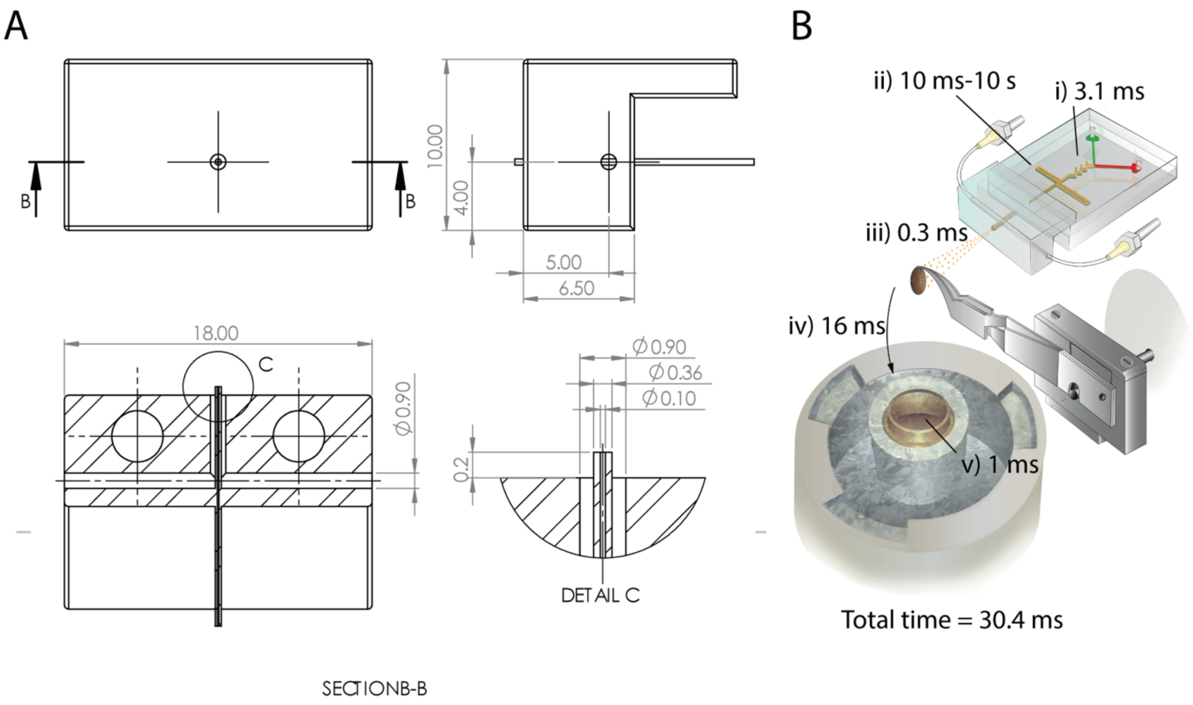

Supplementary Figure 3.

A Technical drawing of the nozzle. Dimensions in mm **B** Overview of time to generate one grid on the system. i) Time for mixing two samples ii) variable incubation time iii) Time-of-flight of the droplets in the air iv) Time of tweezers plunging through the air v) Time for water in the sample to transition into vitreous ice. The errors associated with each individual step are minimal compared to overall reaction time.

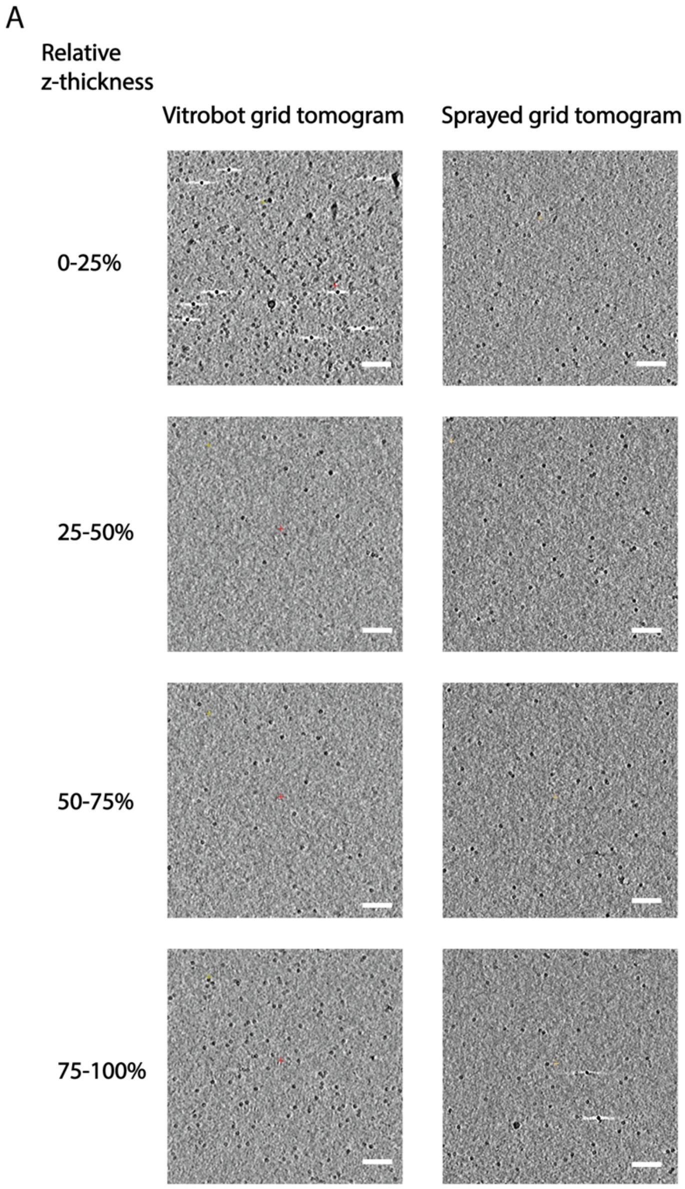

Supplementary Figure 4.

A Slices through tomographic reconstructions at indicated relative depths. Scale bars are 100 nm.

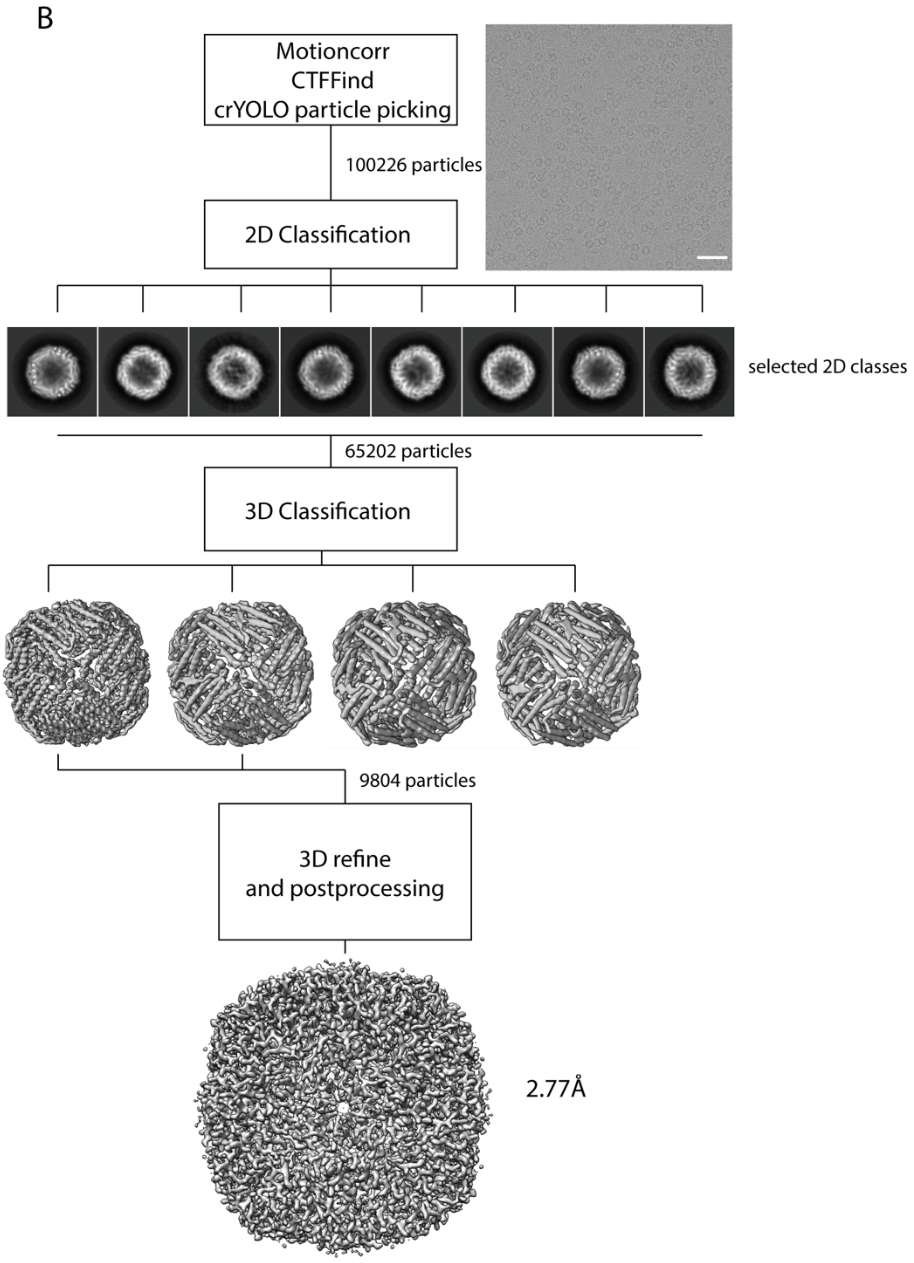

Supplementary Figure 4.

B Workflow of single-particle analysis and statistics of apoferritin from a sample prepared by blot-free spray-plunging. Scale bar 50 nm.

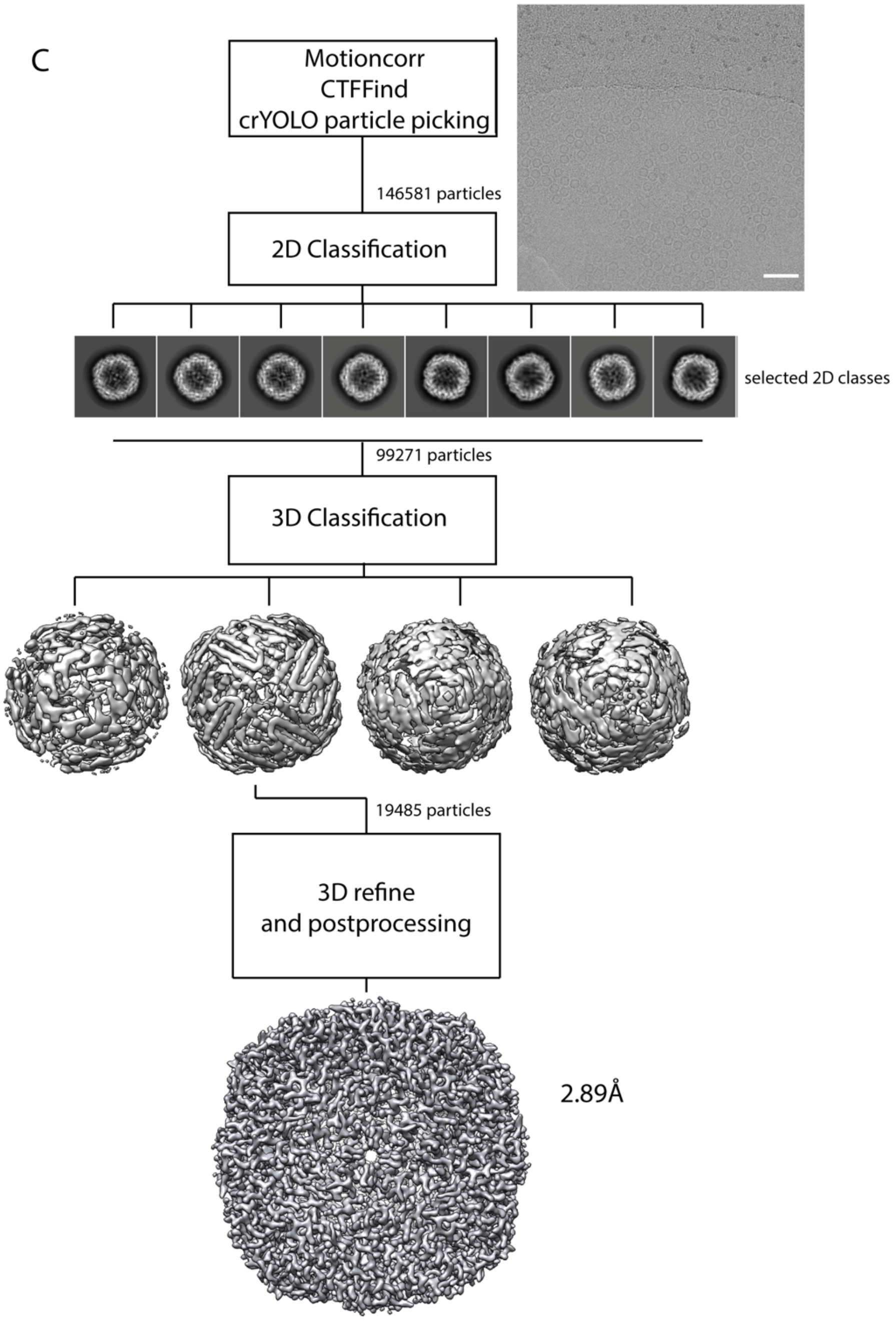

Supplementary Figure 4.

C Workflow of single-particle analysis and statistics of apoferritin from a sample prepared by Vitrobot.

Scale bar 50 nm.

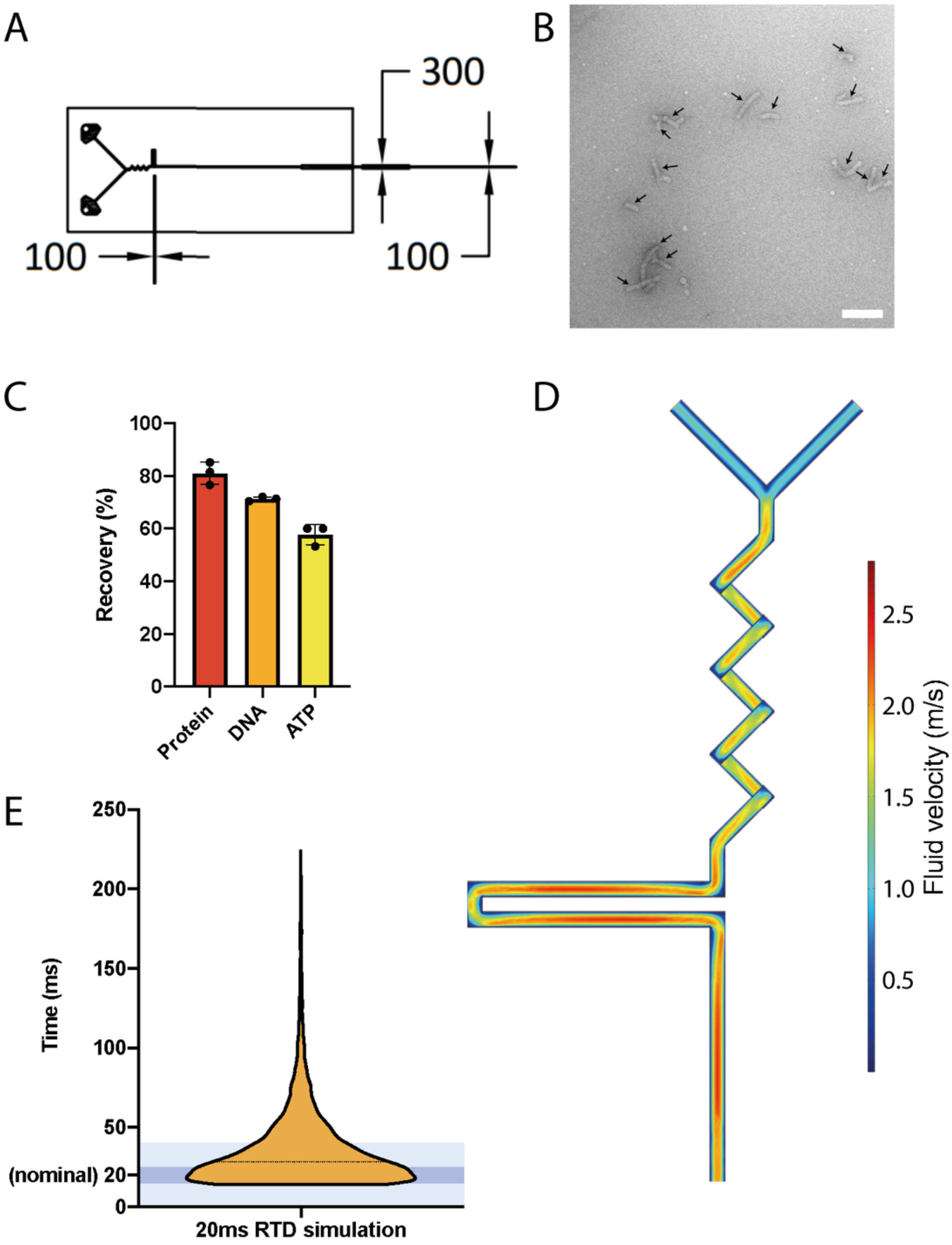

Supplementary Figure 5.

A Technical drawing of the chip with a 20 ms delay line used for Figure 5C (dimensions in µm). **B** Negative stain grid example produced using trEM. RecA-ssDNA filaments indicated by arrows. Negative stain can be used as a suitable alternative to freezing in ethane, as it can stop biological reactions in as little as 10 ms, with the caveat that most high-resolution conformational information is lost[1]. Conversely, it is well suited for initial proof of concept screening due its ease of use. The workflow for producing such samples was identical to the one described for cryo conditions, except for substituting the ethane cup for a custom-made plastic cup which contains 500 µl of 2% uranyl acetate. **C** Quantification of sample loss to the PDMS chip. Source data are provided as a Source Data file. **D** Velocity of flow through the chip, which is computationally simulated using the conditions shown in **Figure 5C** CFD simulation of flow through the chip in **Figure 5C. E** Histogram of the simulated residence time distribution. Dark blue is 15-25 ms and contains 33% of total data. Light blue is 0-40 ms and contains 70% of total data. Source data are provided as a Source Data file.

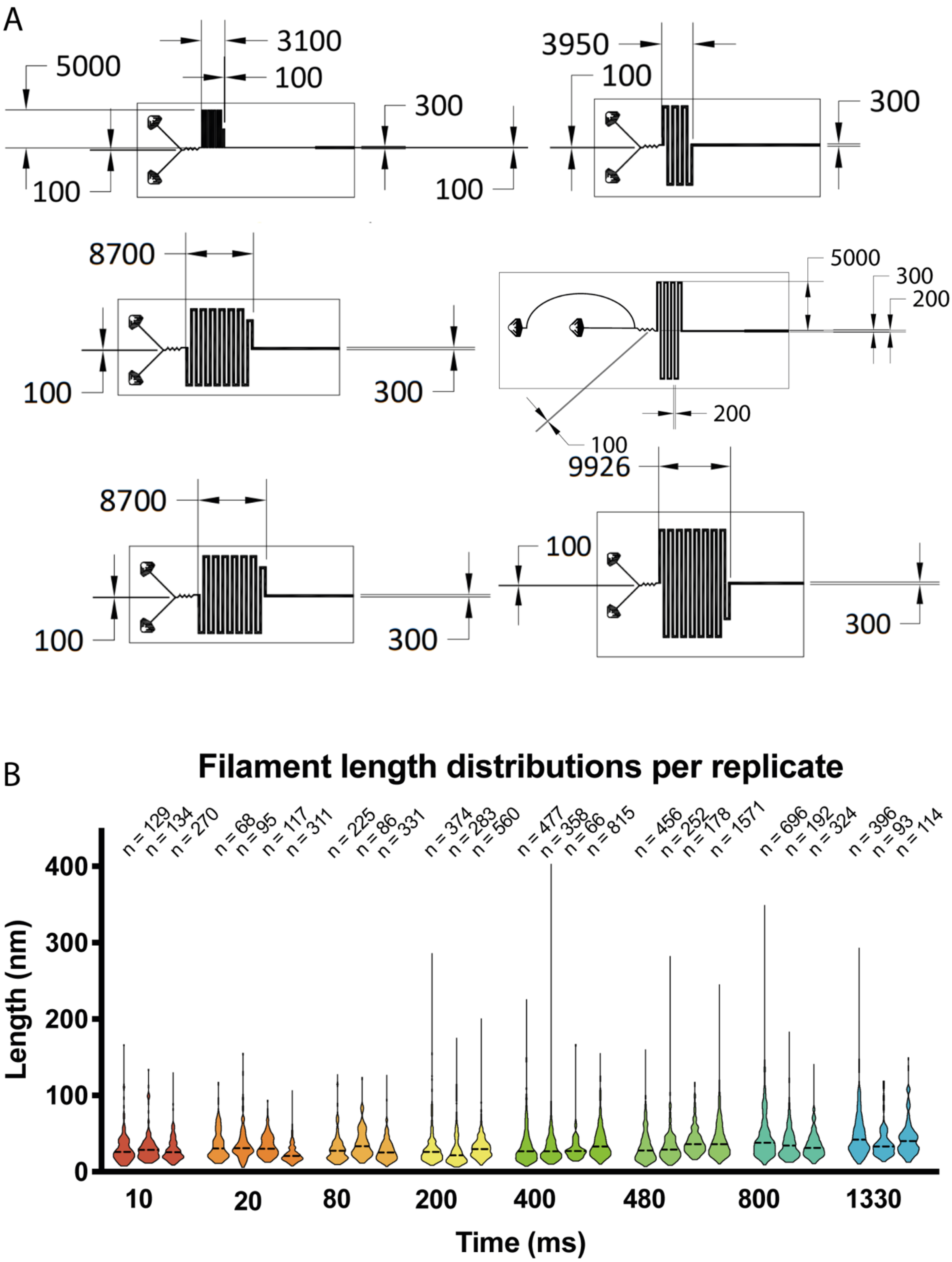

Supplementary Figure 6.

A Technical drawings of microfluidic chips and delay line designs used for producing the data in Figure 6A (dimensions in µm). Z-height of delay line 100 µm in first three panels, 200 µm in last three panels. **B** Violin plots of individual data points whose medians were averaged for the growth curve shown in Figure 6B**.** Source data are provided as a Source Data file.

Supplementary references

1. Zhao, F.-Q. & Craig, R. Capturing time-resolved changes in molecular structure by negative staining. *J. Struct. Biol.* **141**, 43–52 (2003).
