# Supplementary material for: Modular microfluidics enables kinetic insight from time-resolved cryo-EM
Source: Nat Commun. 2020 Jul 10;11:3465. doi: 10.1038/s41467-020-17230-4 (PMC7351747; doi:10.1038/s41467-020-17230-4)
Supplement: Supplementary file 2 — Description of Additional Supplementary Files [file 41467_2020_17230_MOESM2_ESM.pdf]

Description of Additional Supplementary Files

1. **Supplementary data 1 -** Source Code of the software running the device and its graphics user interface.
2. **Supplementary data 2** 3D CAD model of the microfluidics chip for mixing and 10 milliseconds incubation.
3. **Supplementary data 3** 3D CAD model of the microfluidics chip for mixing and 20 milliseconds incubation.
4. **Supplementary data 4** 3D CAD model of the microfluidics chip for mixing and 80 milliseconds incubation.
5. **Supplementary data 5** 3D CAD model of the microfluidics chip for mixing and 200 milliseconds incubation.
6. **Supplementary data 6** 3D CAD model of the microfluidics chip for mixing and 400 milliseconds incubation.
7. **Supplementary data 7** 3D CAD model of the microfluidics chip for mixing and 480 milliseconds incubation.
8. **Supplementary data 8** 3D CAD model of the microfluidics chip for mixing and 800 milliseconds incubation.
9. **Supplementary data 9** 3D CAD model of the microfluidics chip for mixing and 1330 milliseconds incubation.
10. **Supplementary data 10** Technical drawing for workshop production of the motor and gear mechanism mount.
11. **Supplementary data 11** Detailed view of the technical drawing for workshop production of the motor and gear mechanism mount.
12. **Supplementary movie 1** High-speed camera video of the sprayed sample at 0.8 Bar hitting the grid while plunging.
13. **Supplementary movie 2** High-speed camera video of the sprayed sample at 0.1 Bar hitting the grid while plunging.
