# Supplementary material for: Modular microfluidics enables kinetic insight from time-resolved cryo-EM
Source: Nat Commun. 2020 Jul 10;11:3465. doi: 10.1038/s41467-020-17230-4 (PMC7351747; doi:10.1038/s41467-020-17230-4)
Supplement: Supplementary file 3 — Supplementary Data 1 [file 41467_2020_17230_MOESM3_ESM.rtf]

import cc.arduino.*; //Library to control arduino with processingimport org.firmata.*; //Library to interface with Firmata, the relay code running on the arduinoimport controlP5.*; //Library for all the buttons and sliders used in the GUIimport processing.serial.*; //Serial control libraryArduino arduino; //define serial control object "arduino"Serial pump; //define serial control object "pump"ControlP5 cp5; //cp5 Object that controls all of the pushable buttonsControlP5 cp5b; //cp5 Object for Plunge angle sliderControlP5 cp5c; //cp5 Object for Align angle sliderControlP5 cp5d; //cp5 Object for Off angle sliderPFont font; //allows custom fontsint slider = 100; //allows slidersint solstatus = 13; //Indicates the port on the arduino where the wire for the gas is plugged intoint align = 0;//align position variableint angle = 0;//Plunge position variableint off = 0; //off position variableint previous = 0;float fltalign; // float variables to translate output of sliders into input Servo can understandfloat fltangle;float fltoff;void setup(){ //setup code that runs once when program is started  size(2000, 1200); //control panel size(width,height)    println(Arduino.list());  arduino = new Arduino(this, Arduino.list()[1], 57600); //Arduino port here  arduino.pinMode(4, Arduino.SERVO);//"4" signifies the pin on the arduino board where the Servo wire is connected to.  arduino.servoWrite(4, align); // sets default position of Servo to be at the "Align" when program starts, pretty sure this doesnt work right now.  arduino.pinMode(solstatus, Arduino.OUTPUT); //Connects to the gas valve at pin "solstatus"  cp5 = new ControlP5(this);    font = createFont("calibri light", 40);  // custom fonts for button and title  cp5.addButton("Plunge")//all buttons in the user interface with parameters    .setPosition(100,50)    .setSize(300,170)    .setFont(font)   ;    cp5.addButton("Idle")    .setPosition(600,50)    .setSize(50,20)    .setFont(font)   ;   cp5.addButton("Align")    .setPosition(100,250)    .setSize(300,170)    .setFont(font)   ;   cp5.addButton("Off")    .setPosition(100,450)    .setSize(300,170)    .setFont(font)   ;   cp5.addButton("Gas")    .setPosition(100,650)    .setSize(300,170)    .setFont(font)   ;     cp5.addButton("Pump")    .setPosition(200,900)    .setSize(200,200)    .setFont(font)    .setColorBackground(color(67,164,52))   ;    cp5.addButton("Stop_pump")    .setPosition(400,900)    .setSize(200,200)    .setFont(font)    .setColorBackground(color(235,23,38))    .setLabel("Stop")   ;   PImage[] img = {loadImage("Record.jpg")};   cp5.addButton("Reverse_pump")    .setPosition(600,900)    .setSize(200,200)    .setFont(font)    .setLabel("Reverse")    .setImages(img)    .updateSize()   ;cp5b = new ControlP5(this); //These settings for the sliders are copied of the internet, I don't know what all of them do.   Slider a = cp5b.addSlider("plunge angle")     .setBroadcast(false)     .setRange(0, 238)     .setValue(75)     .setPosition(450, 50)     .setSize(300,170)     .setFont(font)     .setSliderMode(Slider.FLEXIBLE)     .setBroadcast(true)     ;cp5c = new ControlP5(this);   Slider b = cp5c.addSlider("Align angle")     .setBroadcast(false)     .setRange(64, 70)     .setValue(65)     .setPosition(450, 250)     .setSize(300,170)     .setFont(font)     .setSliderMode(Slider.FLEXIBLE)     .setBroadcast(true)     ;cp5d = new ControlP5(this);   Slider d = cp5d.addSlider("Off angle")     .setBroadcast(false)     .setRange(0, 238)     .setValue(38)     .setPosition(450, 450)     .setSize(300,170)     .setFont(font)     .setSliderMode(Slider.FLEXIBLE)     .setBroadcast(true)     ;}void draw(){ //Everything in "void draw" runs constantly, might slow down the program.  background(135, 206, 235); //(rgb for the background color)    fill(0, 0, 0);  textFont(font);  text("Time-Resolved Cryo-EM", 50, 30);    int newAngle = constrain(angle,0,180);  fltangle = int(cp5b.getController("plunge angle").getValue()); //saves the value of the slider as fltangle  newAngle = Math.round(fltangle); //rounds the fltangle so that the servo can be sent an integer value for servo positionif(newAngle !=angle){//this makes the servo respond in real time to slider movement  println ("newPos = " +newAngle); //println is a command to report in the black window below what the program is doing for debugging purposes, can be ignored otherwise  arduino.servoWrite(4, constrain(angle / 2, 0, 180));  angle = newAngle; }  int newAlign = constrain(angle,0,180);  fltalign = int(cp5c.getController("Align angle").getValue());  newAlign = Math.round(fltalign);if(newAlign !=align){  println ("newAlig = " +newAlign);  arduino.servoWrite(4, constrain(align / 2, 0, 180));  align = newAlign;  }  int newoff = constrain(off,0,180);  fltoff = int(cp5d.getController("Off angle").getValue());  newoff = Math.round(fltoff);if(newoff !=off){  println ("newOff = " +newoff);  arduino.servoWrite(4, constrain(off / 2, 0, 180));  off = newoff;  }}  void Plunge(){ //if the named button is pushed, reads the corresponding variable from the slider and sends servo to positionarduino.servoWrite(4, constrain(angle / 2, 0, 180)); //"angle" here indicates variable where current slider setting is storedprintln ("on");}void Align(){arduino.servoWrite(4, constrain(align / 2, 0, 180));println ("align");}void Off(){arduino.servoWrite(4, constrain(off / 2, 0, 180));println ("off");}void Gas(){ //when gas button is pushed, turns onprintln("gas");if(previous ==0){  arduino.digitalWrite(solstatus, Arduino.HIGH);    delay(1000);  previous = 1 ;} else{  arduino.digitalWrite(solstatus, Arduino.LOW);  previous = 0;  }}void Pump(){ //Controls for the pump control  pump = new Serial(this, Serial.list()[0], 9600); //opens communication to pump  println("connected");pump.write("irun\r"); //command list that the pump can read is fixed and can be found in the pump manual, must add "\r" for carriage return.  pump.stop(); //closes communication to pumpprintln("running pump");}void Stop_pump(){  pump = new Serial(this, Serial.list()[0], 9600);  println("connected");pump.write("stop\r");pump.stop();println("stopping pump");}void Reverse_pump(){  pump = new Serial(this, Serial.list()[0], 9600);  println("connected");pump.write("rrun\r");pump.stop();println("reversing pump");}void idle(){pump.stop();println("idle");}
