# Supplementary material for: Modular microfluidics enables kinetic insight from time-resolved cryo-EM
Source: Nat Commun. 2020 Jul 10;11:3465. doi: 10.1038/s41467-020-17230-4 (PMC7351747; doi:10.1038/s41467-020-17230-4)
Supplement: Supplementary file 12 — Supplementary Data 10 [file 41467_2020_17230_MOESM12_ESM.pdf]

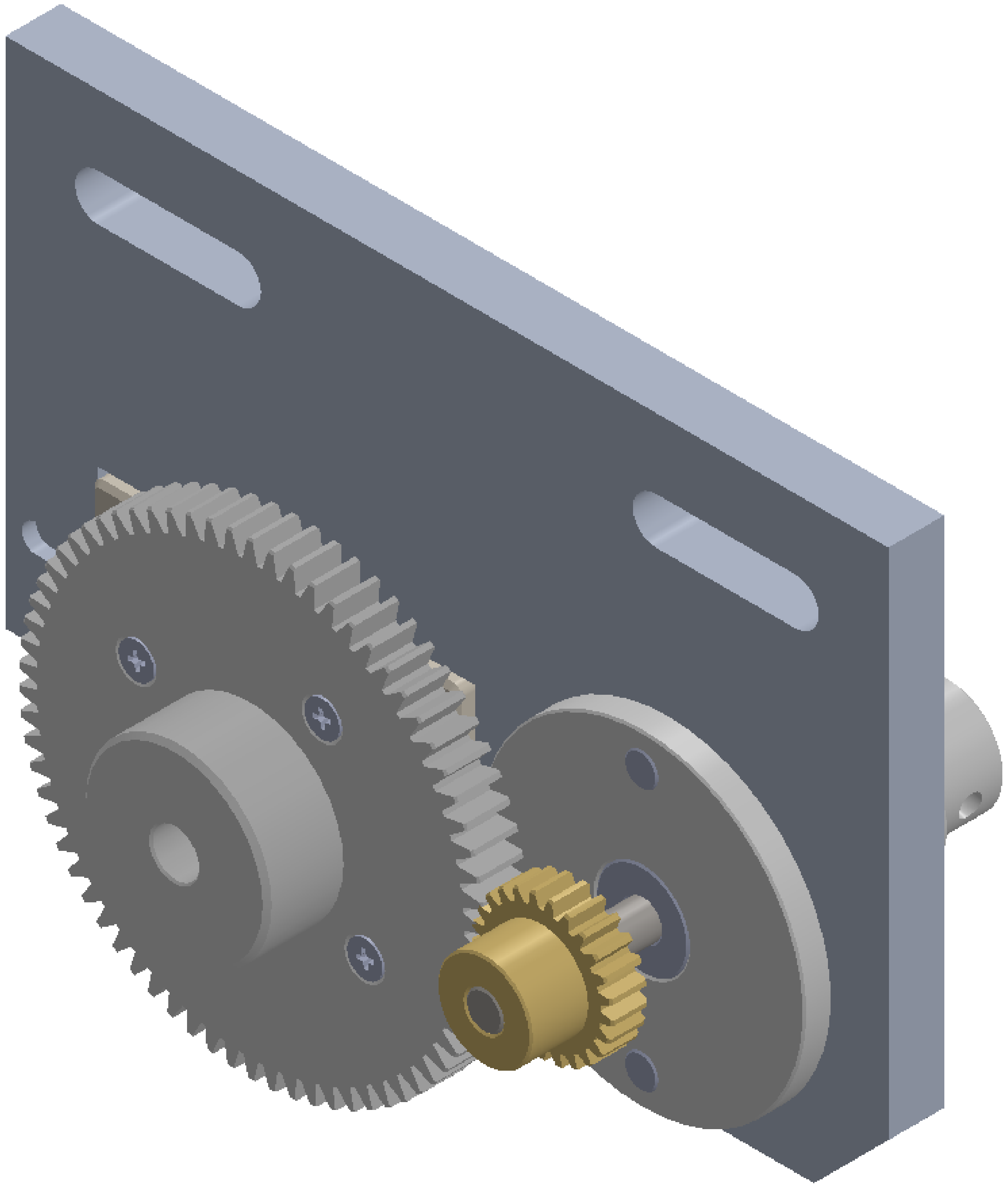

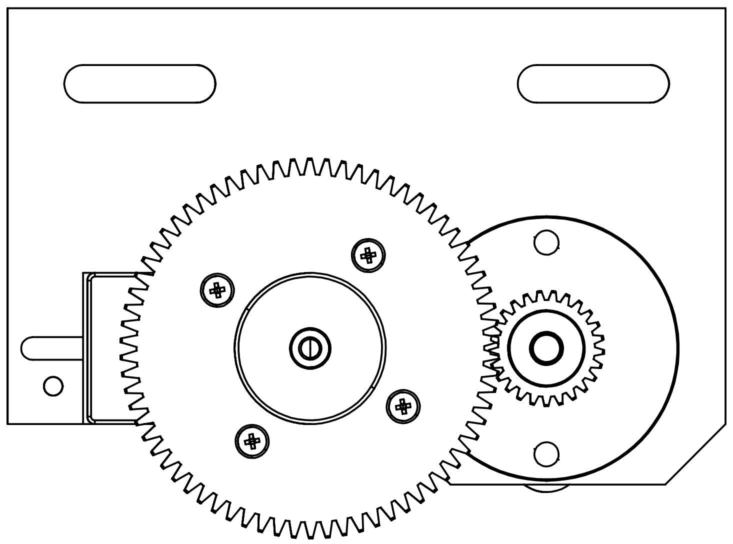

UNLESS OTHERWISE SPECIFIED:<br>DIMENSIONS ARE IN MILLIMETERS<br>SURFACE FINISH:<br>TOLERANCES:<br>LINEAR:<br>ANGULAR:	FINISH:	DEBURR AND<br>BREAK SHARP<br>EDGES	DO NOT SCALE DRAWING / REVISION

	NAME	SIGNATURE	DATE
DRAWN	Nasr Esfahani		
CHK'D			
APPV'D			
MFG			
Q.A			

MATERIAL:

WEIGHT:

TITLE:

Gear mechanism (old system)

DWG NO.

A3

SCALE:1:1

SHEET 1 OF 6

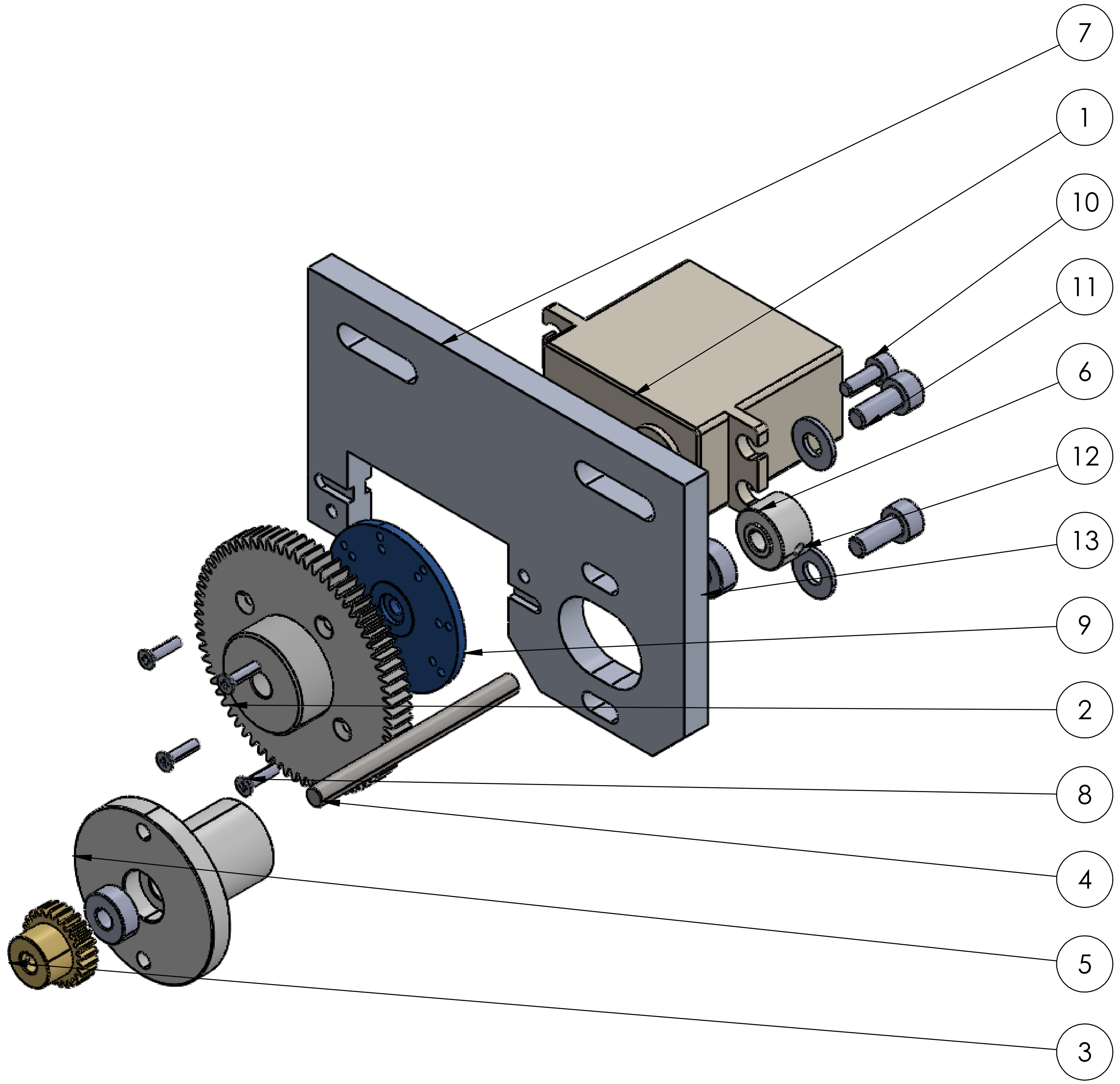

ITEM NO.	DESCRIPTION	QTY.
1	KST Servo (Highest hg800 digital)	1
2	Gear 70T	1
3	Gear 20T	1
4	Bearing shaft	1
5	Bearing block	1
6	End cap	1
7	Servo mounting plate.step	1
8	Countersunk flat head DIN EN ISO 7064-1-M2x8.step	4
9	Servo horn.step	1
10	Socket head cap screw-DIN 912 M3x8.step	2
11	Socket head cap DIN912 M4x10.step	2
12	Plain washer 6902 DIN6902A4.55.step	2
13	Bearing standard	2

UNLESS OTHERWISE SPECIFIED:
DIMENSIONS ARE IN MILLIMETERS
SURFACE FINISH:
TOLERANCES:
LINEAR:
ANGULAR:

FINISH:

DEBURR AND BREAK SHARP EDGES

DO NOT SCALE DRAWING

REVISION

	NAME	SIGNATURE	DATE
DRAWN	Nasr Esfahani		
CHK'D			
APPV'D			
MFG			
Q.A			

MATERIAL:

WEIGHT:

TITLE: Exploded assembly

DWG NO. Gear mechanism (old system)

A3

SCALE:1:2

SHEET 2 OF 6

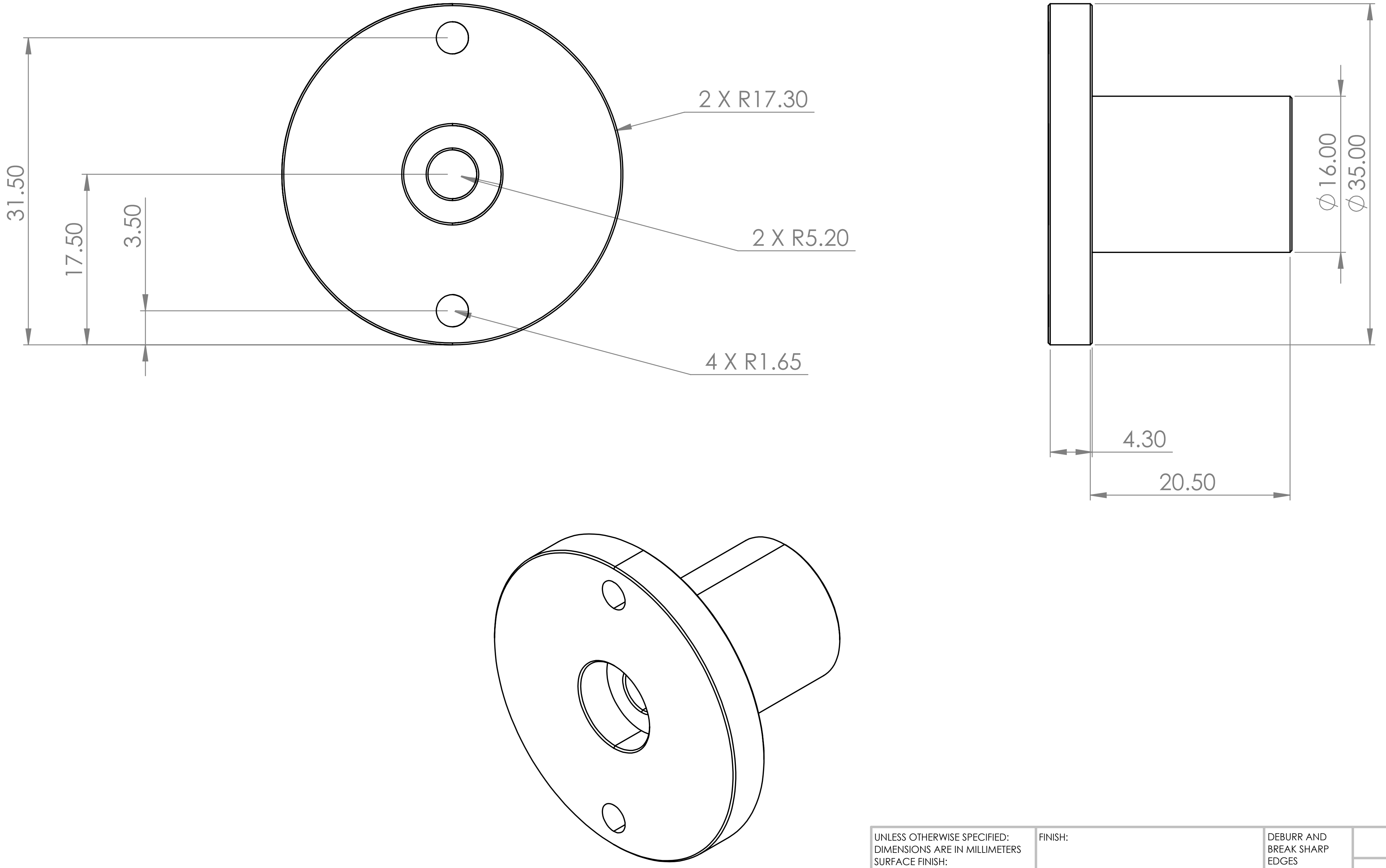

UNLESS OTHERWISE SPECIFIED:
DIMENSIONS ARE IN MILLIMETERS
SURFACE FINISH:
TOLERANCES:
LINEAR:
ANGULAR:

FINISH:

DEBURR AND BREAK SHARP EDGES

DO NOT SCALE DRAWING

REVISION

	NAME	SIGNATURE	DATE
DRAWN	Nasr Esfahani		
CHK'D			
APPV'D			
MFG			
Q.A			

MATERIAL:

WEIGHT:

TITLE:

Bearing block

DWG NO.

Gear mechanism (old system)

A3

SCALE:2:1

SHEET 3 OF 6

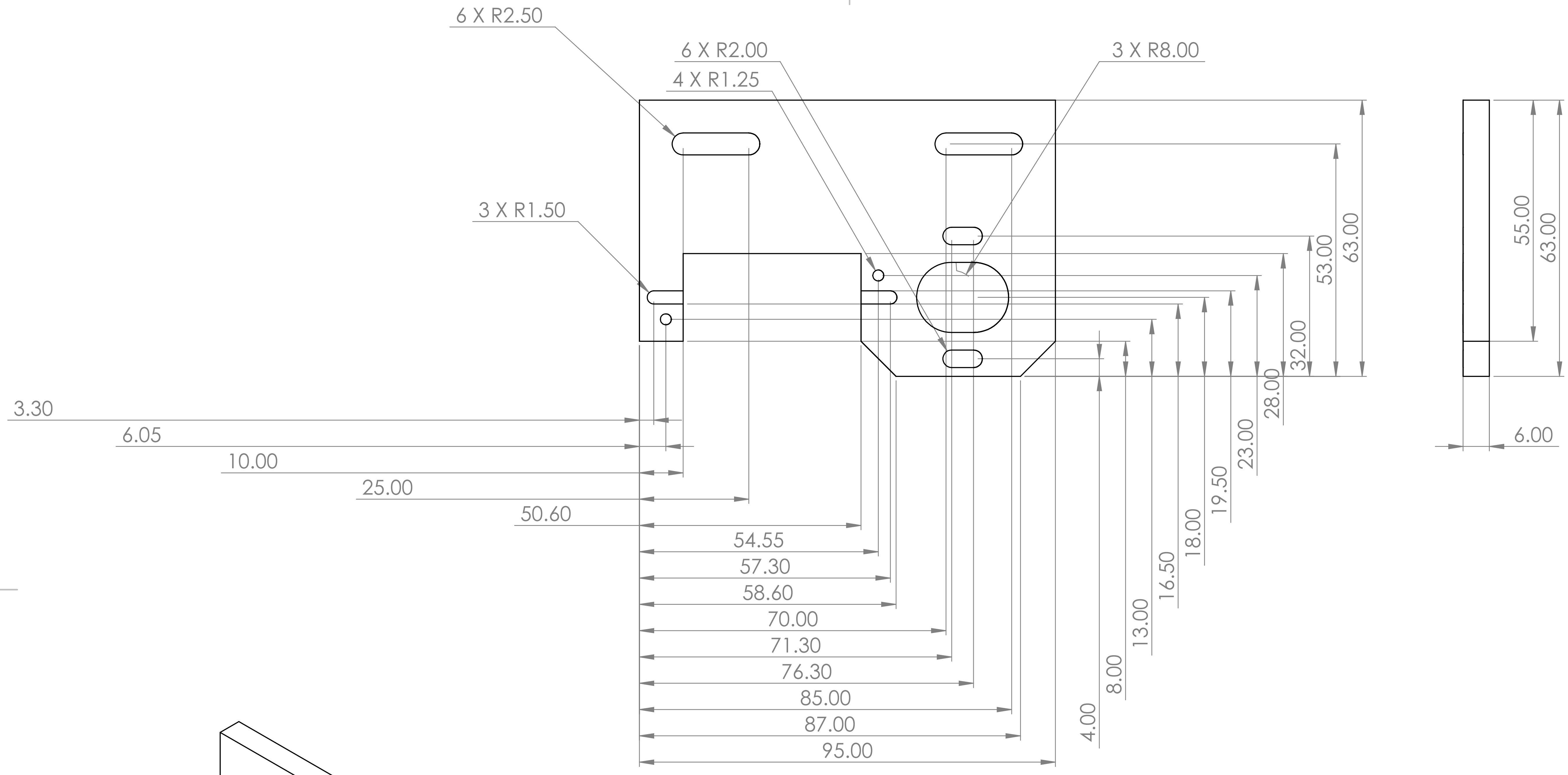

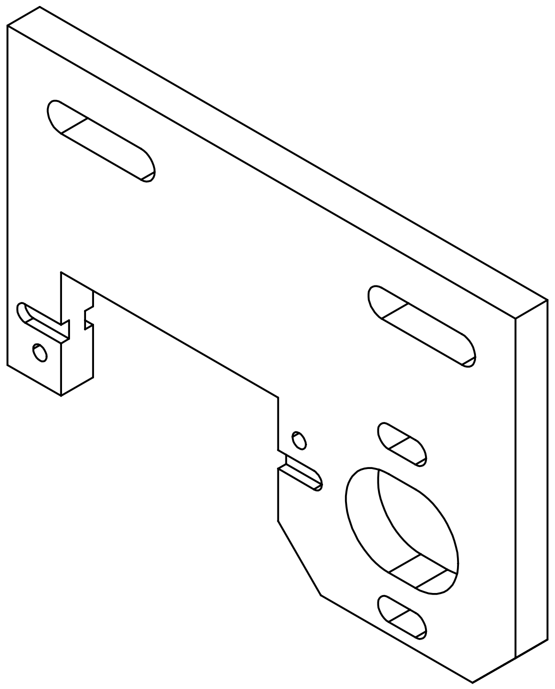

UNLESS OTHERWISE SPECIFIED:
DIMENSIONS ARE IN MILLIMETERS
SURFACE FINISH:
TOLERANCES:
LINEAR:
ANGULAR:

FINISH:

DEBURR AND BREAK SHARP EDGES

DO NOT SCALE DRAWING

REVISION

	NAME	SIGNATURE	DATE
DRAWN	Nasr Esfahani		
CHK'D			
APPV'D			
MFG			
Q.A			

MATERIAL:

WEIGHT:

TITLE:

Servo mounting holder

DWG NO.

Gear mechanism (old system)

A3

SCALE:2:1

SHEET 4 OF 6

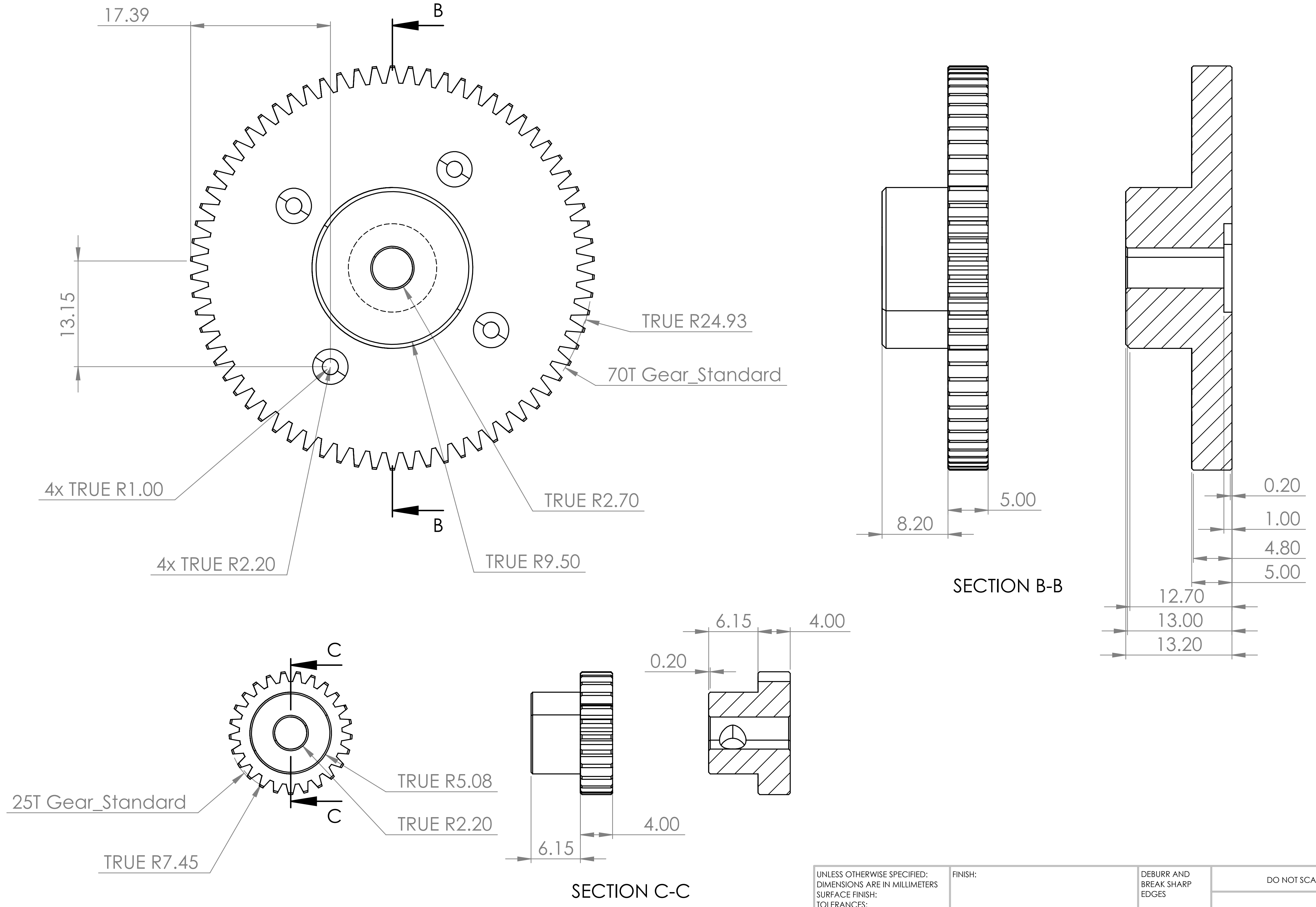

UNLESS OTHERWISE SPECIFIED: DIMENSIONS ARE IN MILLIMETERS SURFACE FINISH: TOLERANCES: LINEAR: ANGULAR:	FINISH:	DEBURR AND BREAK SHARP EDGES	DO NOT SCALE DRAWING	REVISION

	NAME	SIGNATURE	DATE		
DRAWN	Nasr Esfahani				TITLE: Gears (70T and 25T)
CHK'D					
APPV'D					
MFG					
Q.A				MATERIAL:	DWG NO. Gear mechanism (old system) — A3
				WEIGHT:	SCALE:2:1 — SHEET 5 OF 6

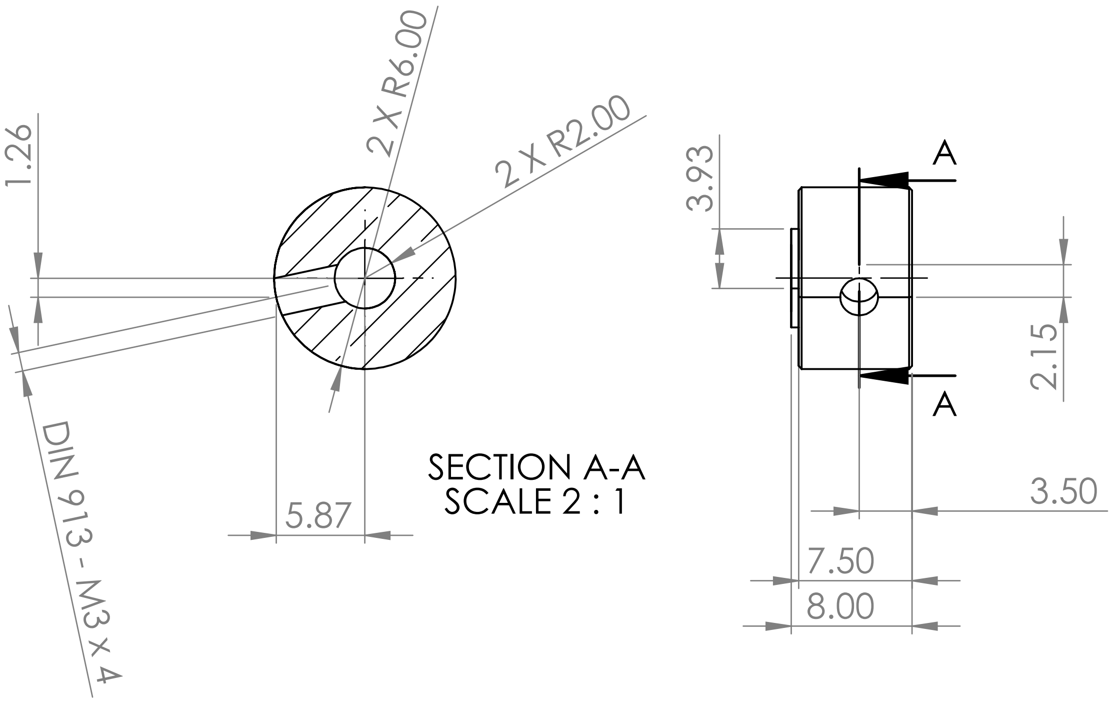

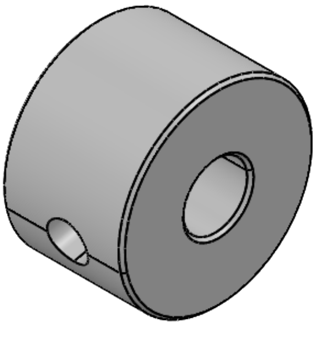

UNLESS OTHERWISE SPECIFIED: DIMENSIONS ARE IN MILLIMETERS SURFACE FINISH: TOLERANCES: LINEAR: ANGULAR:		FINISH:		DEBURR AND BREAK SHARP EDGES	DO NOT SCALE DRAWING	REVISION
	NAME	SIGNATURE	DATE		TITLE:	
DRAWN	Nasr Esfahani				End cap	
CHK'D						
APPV'D						
MFG						
Q.A				MATERIAL:	DWG NO. Gear mechanism (old system)	A4
				WEIGHT:	SCALE:5:1	SHEET 6 OF 6
