# Supplementary figures and images for: Modular microfluidics enables kinetic insight from time-resolved cryo-EM
Source: Nat Commun. 2020 Jul 10;11:3465. doi: 10.1038/s41467-020-17230-4 (PMC7351747; doi:10.1038/s41467-020-17230-4)

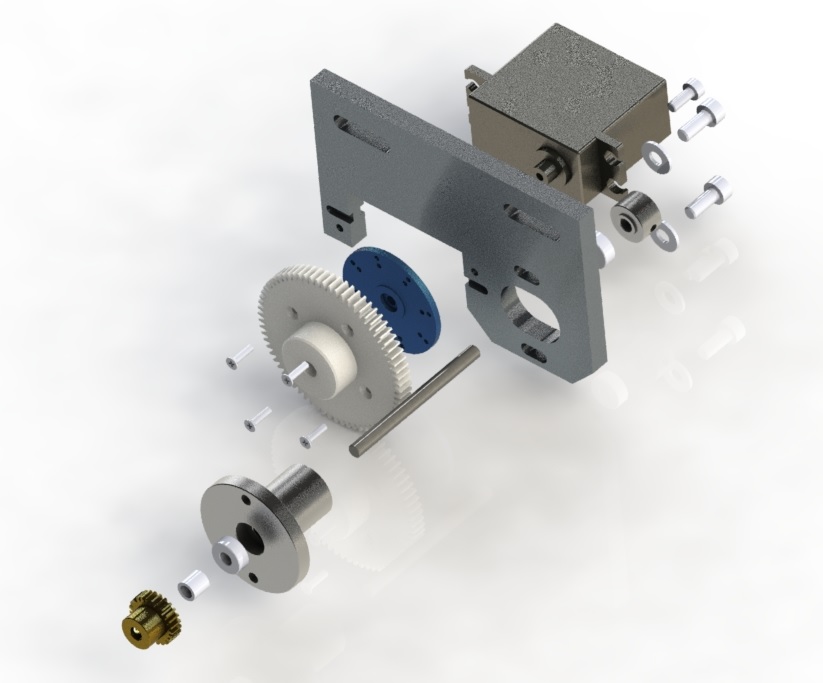

Supplement: Supplementary file 13 — Supplementary Data 11 [file 41467_2020_17230_MOESM13_ESM.jpg]
