# Supplementary material for: Modular microfluidics enables kinetic insight from time-resolved cryo-EM
Source: Nat Commun. 2020 Jul 10;11:3465. doi: 10.1038/s41467-020-17230-4 (PMC7351747; doi:10.1038/s41467-020-17230-4)
Supplement: Supplementary file 16 — Reporting Summary [file 41467_2020_17230_MOESM16_ESM.pdf]

nature research

Corresponding author(s): Radoslav Enchev

Last updated by author(s): Jun 4, 2020

Reporting Summary

Nature Research wishes to improve the reproducibility of the work that we publish. This form provides structure for consistency and transparency in reporting. For further information on Nature Research policies, see Authors & Referees and the Editorial Policy Checklist .

Statistics

For all statistical analyses, confirm that the following items are present in the figure legend, table legend, main text, or Methods section.

n/a	Confirmed	
☐	☒	The exact sample size (*n*) for each experimental group/condition, given as a discrete number and unit of measurement
☐	☒	A statement on whether measurements were taken from distinct samples or whether the same sample was measured repeatedly
☒	☐	The statistical test(s) used AND whether they are one- or two-sided<br>*Only common tests should be described solely by name; describe more complex techniques in the Methods section.*
☒	☐	A description of all covariates tested
☒	☐	A description of any assumptions or corrections, such as tests of normality and adjustment for multiple comparisons
☐	☒	A full description of the statistical parameters including central tendency (e.g. means) or other basic estimates (e.g. regression coefficient) AND variation (e.g. standard deviation) or associated estimates of uncertainty (e.g. confidence intervals)
☒	☐	For null hypothesis testing, the test statistic (e.g. *F*, *t*, *r*) with confidence intervals, effect sizes, degrees of freedom and *P* value noted<br>*Give P values as exact values whenever suitable.*
☒	☐	For Bayesian analysis, information on the choice of priors and Markov chain Monte Carlo settings
☒	☐	For hierarchical and complex designs, identification of the appropriate level for tests and full reporting of outcomes
☒	☐	Estimates of effect sizes (e.g. Cohen's *d*, Pearson's *r*), indicating how they were calculated

Our web collection on statistics for biologists contains articles on many of the points above.

Software and code

Policy information about availability of computer code

Data collection	Arduino 1.8.7 (Firmata), Processing 3.4(ControlP5 library, processing.serial library, arduino library, firmata library), PFV 4.0.1.0, EPU 2.6, COMSOL 5.4, Tomography 4.0.
Data analysis	FIJI 2.0.0, VisiSize 6.5.39, RELION 3.0, Scipion 1.2.1 and 2.0, CTFFind4, Motioncor2, crYOLO 1.3.0, PRISM 8.0.3, Excel 16.16.3, Matlab 9.4, IMOD 4.9.8, MolMatch, Amira 5.3, cryoSPARC 2.15

For manuscripts utilizing custom algorithms or software that are central to the research but not yet described in published literature, software must be made available to editors/reviewers. We strongly encourage code deposition in a community repository (e.g. GitHub). See the Nature Research guidelines for submitting code & software for further information.

Data

Policy information about availability of data

All manuscripts must include a data availability statement. This statement should provide the following information, where applicable:

- Accession codes, unique identifiers, or web links for publicly available datasets
- A list of figures that have associated raw data
- A description of any restrictions on data availability

EM maps are deposited in the EM Data Bank under EMD-10712 (trEM) and EMD-10714 (Vitrobot). Technical drawings and other data are available from the corresponding authors upon reasonable request.

Field-specific reporting

Please select the one below that is the best fit for your research. If you are not sure, read the appropriate sections before making your selection.

Life sciences study design

All studies must disclose on these points even when the disclosure is negative.

Sample size	Sample size of cryo-EM data for single-particle analysis (apoferritin data) were in accordance with usual numbers of molecular images required to achieve near-atomic resolution. RecA-ssDNA filament length measurement experiments were designed as indipendent triplicates (at least) and sample sizes of number of filaments per replicate exceeded 68, as it was empirically determined that a random subset of 50 filaments is statistically representative of the overall population size in such an experiment. Tomography data for apoferritin distribution across the z-direction of holes was quantified from three independent replicates (grids) of either vitrobot or sprayed preparation and up to five holes were analysed per replicate to generate the distribution graphs and error bars in Fig 4 E&G.
Data exclusions	Data were excluded during single particle analysis and high resolution 3D structure determination and refinement in accordance with standard procedures in the field and as explicitly documented in Supp Fig 4 B and C. Data were also excluded during quantification of confocal fluorescent microscopy data assessing mixing efficiency (Fig 2 and Supp Fig 2) by imposing a central region of interest window (10 pixel by 10 pixel) to exclude inevitable deposition of excess fluorescent dye at the edges of the PDMS microfluidic channel as explicitly stated in the Methods section.
Replication	Replicates were done as indicated under sample size above. In particular data collected and analysed for RecA-ssDNA filament length (Fig 6 and Supp Fig 6) were collected in two different labs on two different replicas of the set up (ETH Zurich and Francis Crick Institute in London) over the span of a year.
Randomization	not done because not relevant
Blinding	not done because not relevant

Reporting for specific materials, systems and methods

We require information from authors about some types of materials, experimental systems and methods used in many studies. Here, indicate whether each material, system or method listed is relevant to your study. If you are not sure if a list item applies to your research, read the appropriate section before selecting a response.

Materials & experimental systems

n/a	Involved in the study
☒	☐ Antibodies
☒	☐ Eukaryotic cell lines
☒	☐ Palaeontology
☒	☐ Animals and other organisms
☒	☐ Human research participants
☒	☐ Clinical data

Methods

n/a	Involved in the study
☒	☐ ChIP-seq
☒	☐ Flow cytometry
☒	☐ MRI-based neuroimaging
